# Regional heterogeneity of spatial-temporal characteristics and meteorological-environmental driving factors of hemorrhagic fever with renal syndrome in China

**DOI:** 10.1016/j.onehlt.2026.101454

**Published:** 2026-05-20

**Authors:** Chuanlong Cheng, Michael Tong, Yuchen Hu, Qi Gao, Hui Zuo, Yuqi Zhang, Liang Lu, Xiujun Li

**Affiliations:** a Department of Biostatistics, School of Public Health, Cheeloo College of Medicine, Shandong University, Jinan 250012, Shandong, China; b National Centre for Epidemiology and Population Health, The Australian National University, Canberra, Australian Capital Territory 2601, Australia; c Institute of Clinical Trials and Methodology, MRC Clinical Trials Unit, University College London, London WC1V 6LJ, UK; d National Key Laboratory of Intelligent Tracking and Forecasting for Infectious Diseases, National Institute for Communicable Disease Control and Prevention, Chinese Center for Disease Control and Prevention, Beijing 102211, China

**Keywords:** Hemorrhagic fever with renal syndrome, Regional heterogeneity, Epidemic type, Ecological study, Meteorological and environmental factors

## Abstract

**Background:**

Hemorrhagic fever with renal syndrome (HFRS), a major rodent-borne disease in China, exhibits shifting spatial and incidence patterns due to climate change. From a One Health perspective—integrating human, animal, and environmental health—this China-wide survey aims to identify spatiotemporal characteristics of HFRS and assess regional heterogeneity associated with meteorological and environmental factors.

**Methods:**

Using HFRS case data from 2005 to 2022 in China, this study employed a distributed lag non-linear model to examine associations with meteorological factors, and used a generalized additive mixed model to examine associations with environmental factors. The spatiotemporal effects of these factors were further analyzed across different epidemic types, climate zones, and geographical regions to identify key drivers of HFRS risk.

**Results:**

A bimodal seasonal pattern was observed, with HFRS incidence peaking in autumn/winter (*Apodemus*-Type epidemic regions) and spring (*Rattus*-Type epidemic regions). The effects of monthly mean temperature and relative humidity exhibited inverted U-shaped curves, peaking at 7.5 °C and 38%, respectively, with corresponding relative risks of 1.85 (95% CI: 1.35–2.54) and 1.21 (95% CI: 1.04–1.40). HFRS risk initially rose as precipitation increased and then declined, while extreme precipitation and snow depth inhibited HFRS risk. Impervious surface and water body percentages had greater impacts in *Apodemus*-Type epidemic regions, whereas shrubland and wetland percentages had greater impacts in *Rattus*-Type epidemic regions, indicating significant regional differences.

**Conclusion:**

HFRS exhibits geographical clustering and bimodal seasonal pattern in China, driven by regional heterogeneity in host species, meteorological and environmental factors. These spatiotemporal patterns, viewed through a One Health lens, necessitate region-specific interventions to guide precision prevention and early warning nationwide.

## Highlights

• HFRS seasonality in China varied by host: *Apodemus* (autumn) vs *Rattus* (spring).
• Optimal temperature (0.6–11 °C) & humidity (38%) increased HFRS risk.
• Impervious surface was key in *Apodemus* regions; NDVI was key in *Rattus* regions.

## Graphical abstract

**Figure:**
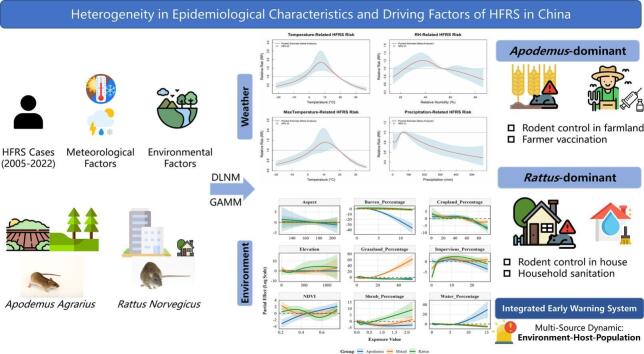

## 1 Introduction

Hemorrhagic fever with renal syndrome (HFRS) is a rodent-borne zoonotic disease caused by hantaviruses (HV) [1]. It is endemic across regions of Asia, Europe, and Africa. Globally, approximately 20,000 HFRS cases occur each year, with about 80% of these cases reported in China [2], [3], [4]. One study indicated that HFRS cases in China were primarily concentrated in the Northeast, Northwest, and Eastern regions [5]. The incidence of HFRS in China exhibits regional specificity and spatio-temporal variability [3], [6], [7]. HFRS epidemic regions have also shown a transition from single-type to mixed-type distributions, influenced by different host species occupying distinct ecological niches [8]. Although overall cases show a recent downward trend, recurrent local outbreaks with “Epidemic-Remission-Recurrence” cycles persist, and HFRS remains a significant public health challenge requiring localized control strategies.

Temperature and precipitation are significantly associated with HFRS risk, affecting host reservoir activity, virus viability, and human exposure [9], [10], [11], [12]. Studies indicate that temperature exhibits non-linear and lagged effects on HFRS incidence, with varying impacts across climatic regions [2], [13]. Similarly, precipitation also shows lagged effects on HFRS transmission [5], [14], [15]. The variations in lagged effects and temperature thresholds are reported across different climate regions. The normalized difference vegetation index (NDVI) serves as an indicator of vegetation density and has been correlated with HFRS incidence, likely reflecting habitat suitability for rodent hosts through the provision of food resources (e.g., seeds, fruits, and plant matter) and shelter [2], [10], [16], [17]. Land use patterns, such as cropland, artificial surfaces, and forested areas, influence rodent population distribution and density by altering food availability, nesting safety, and movement corridors, thereby affecting human–rodent contact rates [10], [17]. The regional variations in land use and vegetation cover may support different rodent species, contributing to differences in HFRS serotype distribution and incidence [10], [17], [18]. Therefore, investigating the meteorological and environmental disparities across various regions could provide valuable insights to support effective HFRS prevention and control strategies.

Despite existing research on meteorological and environmental influences on HFRS, most studies have been confined to single provinces or cities in China, lacking a comprehensive analysis at a national scale. This limitation hinders the understanding of broader epidemiological patterns and the development of tailored intervention strategies [4], [19]. To address this gap, this study aims to: (1) systematically investigate HFRS spatial heterogeneity across regions in China; (2) identify meteorological and environmental factors associated with HFRS risk; and (3) determine key factors in high-incidence regions. The findings will provide scientific evidence and practical guidance for the development of regional specific HFRS prevention and control strategies.

## 2 Methods

### 2.1 Data collection

**HFRS Case Data:** Monthly HFRS case data from 2005 to 2022 were obtained from the China Information System for Disease Control and Prevention (CISDCP), including patient sex, age, address, and date of onset. To better capture the epidemiological characteristics of HFRS in China and ensure model convergence, only cities with cumulative cases greater than 100 during the study period were included. The final included cases accounted for 84.61% of the total cases nationwide.

**Fig. 1:**
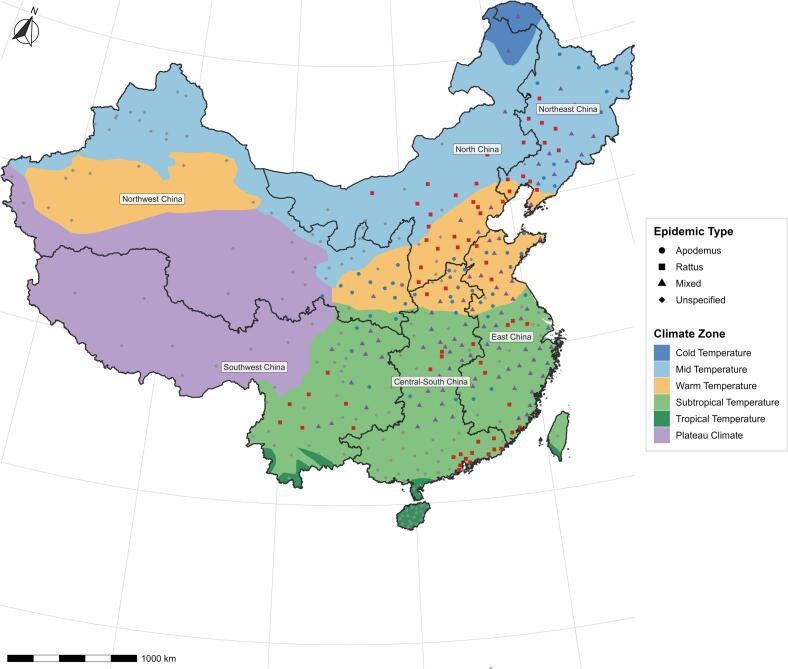
Grouped map of the cities analyzed in this study. **Note:** Grouped map contains information on the epidemic type, climate zone, and geographic region.

HFRS epidemic regions in China can be classified into three types based on the dominant host species and associated virus serotypes: *Apodemus*-Type (Hantaan Virus, HTNV), *Rattus*-Type (Seoul Virus, SEOV), and Mixed-Type (where both types coexist) [19]. To better analyze regional differences, this study classified the data into several groups. Regions were grouped by: HFRS epidemic type regions (*Apodemus*-Type, *Rattus*-Type, Mixed-Type), climate zones (Mid-temperate, Warm-temperate, Subtropical), and geographical regions (Northeast, Northwest, North, East, and Central South China). The grouping map is shown in Fig. 1. The classification of study cities into these groups is detailed in Supplementary Table S1.

**Meteorological Data:** Monthly meteorological data were sourced from the ERA5-Land dataset of the European Centre for Medium-Range Weather Forecasts [20]. ERA5 is the fifth-generation global atmospheric reanalysis dataset from the European Centre for Medium-Range Weather Forecasts (ECMWF), widely recognized as a benchmark product. It assimilates multi-source observations (satellites, radiosondes, ground stations) to produce high-quality gridded data from 1940 to present, with a spatial resolution of 0.25° × 0.25° and hourly temporal resolution. The variables included monthly mean temperature (°C), maximum temperature (°C), minimum temperature (°C), precipitation (mm), relative humidity (%), and snow depth (cm). ERA5 meteorological raster data were collated into monthly data for each city based on the vector map of Chinese cities.

**Environmental Data:** NDVI, elevation, slope, slope orientation, and vegetation coverage (%) were obtained from the National Tibetan Plateau Data Center [21], [22], [23]. Land use data (9 classes) were sourced from “The 30 m annual land cover datasets and its dynamics in China from 1985 to 2023”. The above environmental variables were raster data with a resolution of 30-250 m, and the processing method was the same as that of meteorological factors. The spatial distribution dataset of primary rivers in China was obtained from the Resource and Environment Science and Data Center [24]. The river data was a vector map, and the river distance was calculated from the city center to the river. Detailed descriptions of the environmental data are provided in Supplementary Table S2. All variables were collated into city-level monthly data.

### 2.2 Statistical analysis

Cumulative case counts and average annual incidence rates were calculated for each province to identify high-incidence provinces in China. Additionally, annual and monthly incidence rates across different regions were visualized.

**Part 1 Meteorological factors:** To assess the impact of meteorological factors on HFRS incidence, a two-stage analytical approach was employed. In the first stage, distributed lag non-linear models (DLNM) were fitted for each city to capture the potentially non-linear and delayed effects of monthly meteorological variables on HFRS risk [4], [6]. In the second stage, a random-effects multivariate meta-analysis using restricted maximum likelihood (REML) was performed to obtain pooled effects across cities within different regions [25]. Furthermore, the impact of meteorological factors on HFRS in different regions was estimated. Using the 50th percentile (P_50_) of the overall monthly meteorological factor exposure distribution as the reference, the cumulative non-linear and lagged effects at different exposure levels were estimated. Corresponding relative risk (RR) and 95% confidence interval (CI) were calculated, and cumulative exposure-response curves were plotted.

**Part 2 Environmental factors:** The impact of environmental factors on HFRS incidence was explored using a generalized additive mixed model (GAMM) to identify habitat characteristics in different regions, such as local NDVI and land use [5], [7], [26]. GAMM were constructed for the entire country and then separately for different regions. A model including city-level random effects was built to adjust for the influence of sampling variations among cities. Prior to model fitting, variable selection was performed based on Spearman correlation analysis (*r* < 0.7) and the relative importance of variables from boosted regression trees (BRT) models (> 0.05), ensuring inclusion of variables with significant impact on HFRS and low collinearity. Key variables from different regions were included in the GAMM to compare the strength and patterns of environmental factors across regions. The specific model formulas and settings can be found in the Supplementary Methods.

To assess model robustness, sensitivity analyses were conducted at the national level: a) changing the lag period (from 5 to 7 months); b) changing the degrees of freedom (df = 2, 3, 4) in the DLNM; c) changing the spline function (cubic spline and thin plate spline); and d) changing the smoothing parameter (k = 5, 10) in the GAMM.

Statistical analyses were performed using R version 4.3.1 with the ‘dlnm’, ‘splines’, ‘mgcv’, and ‘gamm4’ packages [27]. All tests were two-sided, and *P* < 0.05 was considered statistically significant.

## 3 Results

### 3.1 Epidemiological characteristics of HFRS in China

**Fig. 2:**
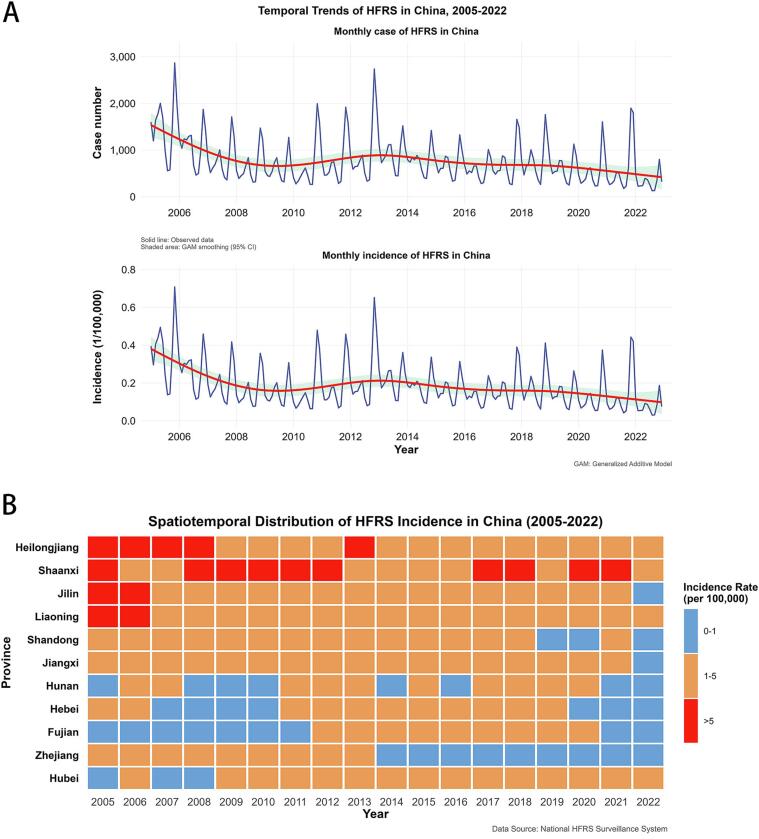
Incidence of HFRS in provinces with high incidence in China from 2005 to 2022. **Note:**Fig. 2A shows the temporal trend of cases and incidence rate of HFRS nationwide. Fig. 2B shows the incidence rates of HFRS in different provinces from 2005 to 2022.

From 2005 to 2022, there were 202,372 HFRS cases reported in China, concentrated in Northeast, Northwest, and East China. Heilongjiang, Shaanxi, Jilin, Liaoning, and Shandong provinces had the highest cumulative cases and annual incidences (Supplementary Table S3). Nationwide cases showed a fluctuating decline characterized by dual annual peaks, with a primary seasonal surge in autumn/winter, followed by a secondary increase in spring (Fig. 2A). The higher incidence rate mainly occurred in Heilongjiang Province, Shaanxi Province, Jilin Province and Liaoning Province (Fig. 2B).

### 3.2 Impact of meteorological factors on HFRS

**Fig. 3:**
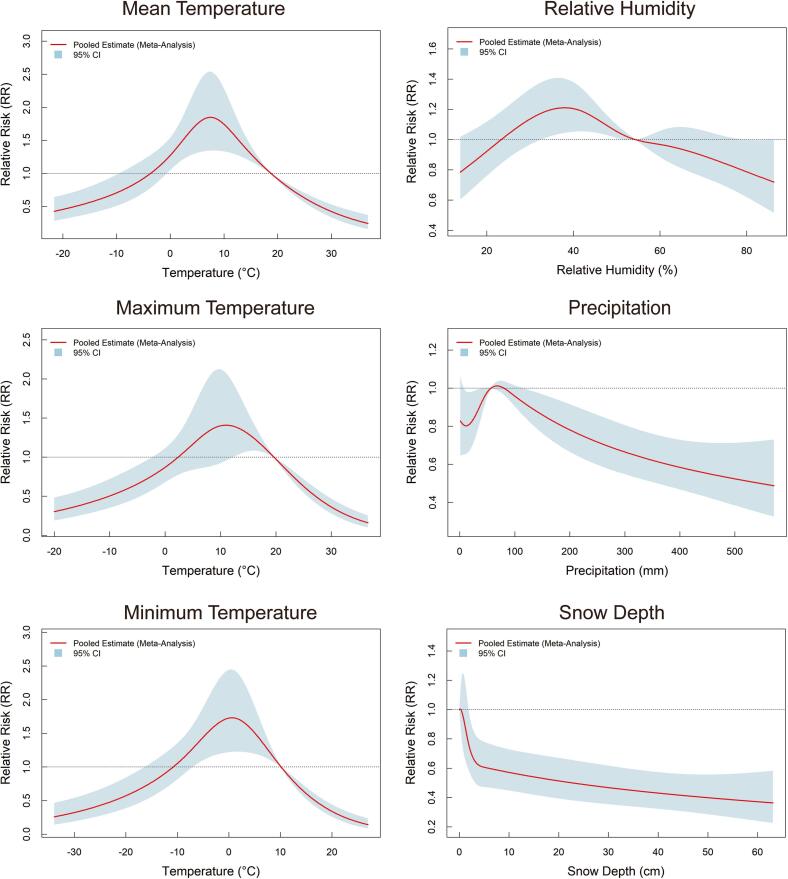
Nonlinear effects of meteorological factors on HFRS. **Note:** Reference values: Mean temperature: 18.8 °C; Maximum temperature: 19.6 °C; Minimum temperature: 10.1 °C; Relative humidity: 54.4%; Precipitation: 57 mm; Snow depth: 0 cm.

The impact of meteorological factors on HFRS risk is presented in Fig. 3. The associations between temperatures and HFRS risk exhibited inverted U-shaped relationships. The strongest effects were observed at 7.5 °C for monthly mean temperature, 11 °C for monthly maximum temperature, and 0.6 °C for monthly minimum temperature, with the corresponding RRs of 1.85 (95% CI: 1.35–2.54), 1.41 (95% CI: 0.96–2.07), and 1.73 (95% CI: 1.22–2.45), respectively. The effect of relative humidity also showed an inverted U-shaped relationship, peaking at 38%, with an RR of 1.21 (95% CI: 1.04–1.40). HFRS risk increased with monthly precipitation up to 68 mm, then declined as precipitation continued to rise with RR of 0.78 (95% CI: 0.67–0.92) at 200 mm. In contrast, snow depth showed a significant negative association with HFRS risk, with RR of 0.57 (95% CI: 0.45–0.73) at 10 cm. Additionally, the effects of meteorological factors were most pronounced at lag of 2–4 months (Supplementary Fig. S7).

**Fig. 4:**
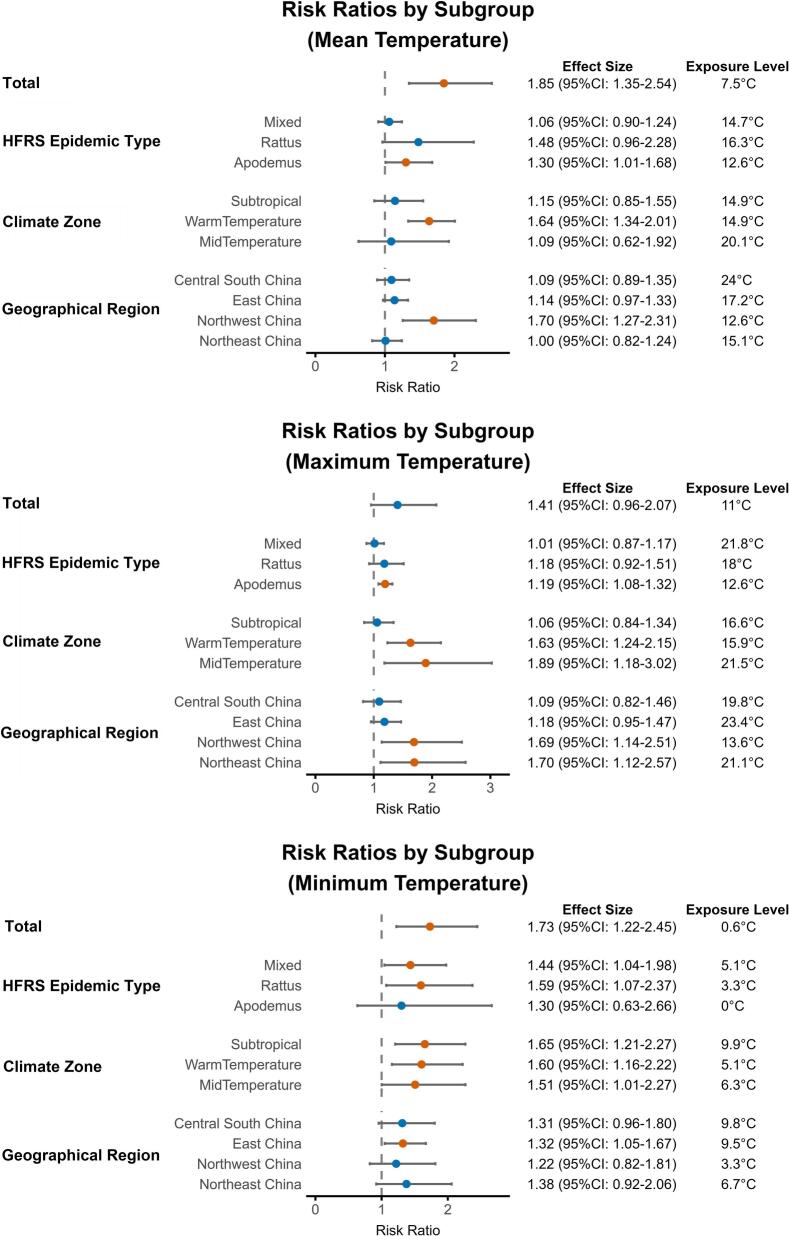
Differences in effect of mean, maximum and minimum temperature across HFRS epidemic types, climate zones, and geographical regions. **Note:** Red dots represent statistically significant results, and blue dots represent results that are not statistically significant.

There were differences in the effects of meteorological factors across HFRS epidemic types, climate zones, and geographical regions (Fig. 4, Supplementary Figs. S8–S11). *Apodemus*-Type epidemic regions showed stronger associations with increases in monthly mean temperature and maximum temperature. Conversely, *Rattus*-Type epidemic regions were most significantly affected by relative humidity (RR = 1.95, 95% CI: 1.57–2.43). Moreover, precipitation posed a protective factor in *Rattus*-Type epidemic regions (RR = 0.52, 95% CI: 0.40–0.69), but it was associated with increased risk in *Apodemus*-Type epidemic regions (RR = 1.46, 95% CI: 1.01–2.12). Snow depth showed a stronger negative association with HFRS risk in *Apodemus*-Type epidemic regions (RR = 0.17, 95% CI: 0.11–0.26).

Across climate zones, the maximum temperature was positively correlated with HFRS risk in mid-temperate regions (RR = 1.89, 95% CI: 1.18–3.02). Minimum temperature showed the strongest effect on HFRS risk (RR = 1.65, 95% CI: 1.27–2.21) in subtropical regions (Supplementary Fig. S9). By geographical region, the effect of mean temperature was strongest in Northwest China (RR = 1.70, 95% CI: 1.27–2.31). HFRS risk was positively correlated with precipitation (RR = 1.52, 95% CI: 1.06–2.17) in Northwest China (Supplementary Fig. S10).

### 3.3 Impact of environmental factors on HFRS

**Fig. 5:**
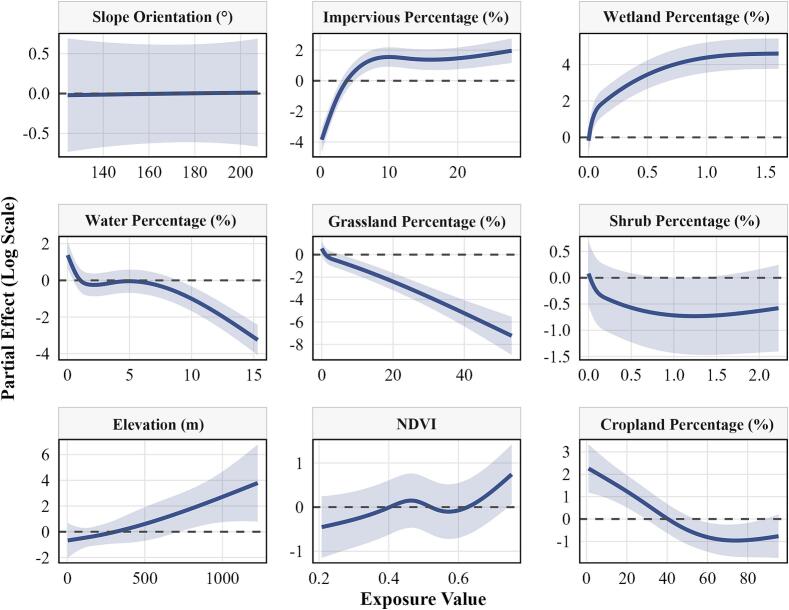
The impact of environmental factors on HFRS. **Note:** NDVI is normalized difference vegetation index.

Based on correlation analysis and BRT model results (Supplementary Figs. S12–S13), the key environmental factors affecting HFRS risks included slope orientation, impervious surface, water body, wetland, shrubland, grassland, elevation, NDVI, and cropland. Results from GAMM effect analysis (Fig. 5) showed that for impervious surfaces, percentages below 10% were negatively associated with HFRS risk, whereas when the percentage exceeded 10%, HFRS risk increased with further expansion of impervious surfaces. Similarly, when wetland percentage was >0 and elevation exceeded 800 m, HFRS risk increased with increasing wetland percentage and elevation. Conversely, when water body percentage was >9% and grassland percentage was >5%, HFRS risk decreased as water body and grassland increased. For cropland, lower percentages were positively associated with HFRS risk, but when the percentage exceeded 40%, HFRS risk decreased with increasing cropland percentage.

**Fig. 6:**
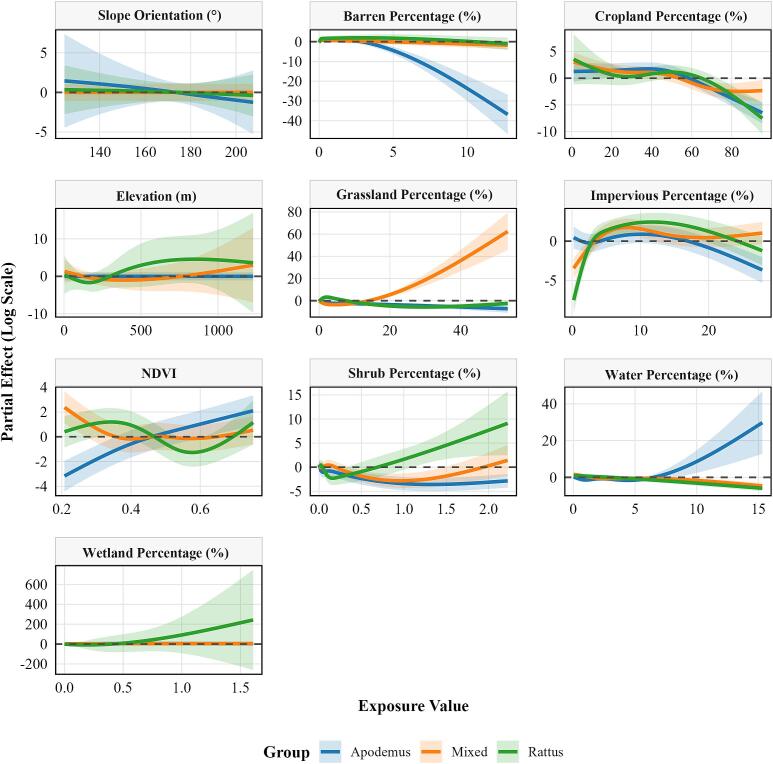
Differences in effect of environmental factors in different epidemic type regions. **Note:** NDVI is normalized difference vegetation index.

The effects of environmental factors on HFRS risk varied significantly across HFRS epidemic-type regions (Fig. 6). In *Apodemus*-Type epidemic regions, HFRS risk increased with lower bare land percentage and higher water body percentage. Additionally, the association between NDVI and HFRS risk was negative at low NDVI levels but became positive with increasing NDVI in *Apodemus*-Type epidemic regions. In *Rattus*-Type epidemic regions, HFRS risk was sensitive to elevation, shrubland, and wetland. In Mixed-Type epidemic regions, HFRS risk increased with increasing grassland percentage (Supplementary Figs. S14–S19).

By climate zone, increasing impervious surface coverage had a negative effect on HFRS risk in mid-temperate regions. Wetland had a significant impact on HFRS risk in warm-temperate regions (Supplementary Figs. S20–S26). By geographical region, cropland and elevation demonstrated greater effects on HFRS risk in Northeast China, while effects of impervious surfaces, NDVI, and elevation were greater in East China. In Northwest China, the HFRS risk was associated with cropland, impervious surfaces, NDVI, and distance to rivers. Details are shown in Supplementary Figs. S27–S35.

The sensitivity analysis indicated that the model results remained stable and robust regardless of adjustments to lag time and degrees of freedom in DLNM, or spline functions and smoothing parameters in GAMM (Supplementary Figs. S36–S37).

## 4 Discussion

This study found that most HFRS cases were primarily concentrated in Northeast China (Heilongjiang, Jilin, and Liaoning provinces), Northwest China (Shaanxi Province), and East China (Shandong Province), exhibiting significant bimodal seasonality. Meteorological factors showed lagged effects on HFRS incidence, with higher precipitation and snow depth negatively associated with HFRS risk. Environmental factors also had impacts on HFRS incidence, which varied across different regions. These findings highlight significant regional differences in epidemic mechanisms, necessitating the development of refined, region-specific prevention and control strategies.

Different epidemic types of HFRS exist across regions in China, which might be due to the distribution of rodent species. For example, *Apodemus agrarius* is widely distributed in Northeast China, whereas both *Apodemus* and *Rattus* species exist in Shaanxi Province in Northwest China [5]. HFRS exhibited distinct seasonal trends across regions: cases peaked during autumn and winter in *Apodemus*-Type epidemic regions, whereas the peak occurred primarily during spring in *Rattus*-Type epidemic regions. Further, HFRS cases exhibited dual peaks during both autumn/winter and spring in Mixed-Type epidemic regions. This is consistent with a previous study that found seasonal patterns were related to the dominant hantavirus genotype, with HTNV primarily spreading in autumn/winter while SEOV spreading in spring [7]. *Apodemus*-Type HFRS cases were widely distributed in higher latitudes, where the host activity periods overlapped with the autumn/winter, whereas *Rattus*-Type HFRS cases were mostly in warm-temperate and subtropical regions, peaking in spring [28]. These differences demonstrate that regional environmental characteristics and rodent species profoundly affect HFRS transmission.

This study found that temperature exhibited an inverted U-shaped effect, with the highest risk occurring within the monthly mean temperature range of 7.5 °C–11 °C, consistent with a previous study in Shaanxi Province [29]. Extremely high or low temperatures are unfavorable for HFRS transmission, suggesting an optimal temperature range that may potentially promote host population growth, virus viability, or increased human-host contact opportunities. A national longitudinal study also highlighted temperature ranges influencing HFRS risk and reported the regional differences associated with host types [30]. Relative humidity also showed an optimal level, potentially favoring virus viability or host activity in the environment [31]. HFRS risk was the highest around 38% relative humidity, particularly in *Rattus*-Type epidemic regions. Precipitation exhibited a “threshold effect” on HFRS, with risk increasing below 68 mm but decreasing above 200 mm. Moderate rainfall likely facilitates vegetation growth, providing ample food and habitat for rodents, thus promoting rodent population growth and increasing HFRS risk. Conversely, excessive rainfall may cause flooding, destroying rodent habitats and reducing HFRS risk [32]. Snow depth showed a significant negative association with HFRS risk, potentially because snow cover restricts rodent activity ranges and reduces human activity in snowy areas. Additionally, the effects of meteorological factors varied across epidemic-type regions, climate zones, and geographical regions. HFRS risk was most sensitive to increases in monthly mean and maximum temperatures in *Apodemus*-Type epidemic regions, warm-temperate climate zone, and Northwest China. This variation is likely related to differences in host species, their breeding cycles, and activity patterns [12], [33], [34], [35], [36], [37], [38]. These regional differences underscore the necessity of considering local meteorological conditions when developing HFRS control strategies.

HFRS risk is also associated with environmental factors. This study found HFRS risk was associated with cropland and water body in *Apodemus*-Type epidemic regions, whereas it was more sensitive to elevation, shrublands, and wetlands in *Rattus*-Type epidemic regions. This is consistent with previous study that found cropland, forestland, and orchards as major factors influencing HFRS distribution, with a negative correlation between HFRS and elevation [39]. Moreover, a higher incidence of HFRS was observed in farmland areas, likely due to the abundant food resources and ideal habitat conditions for rodents, combined with increased human activities during agricultural seasons, which ultimately increased HFRS risk [17], [40], [41]. In comparison, rodent distribution is more restricted in high-altitude regions due to lower temperatures and limited food availability [39]. A threshold effect of impervious surface on HFRS risk was observed: when the percentage remained below 10%, the risk was relatively low due to the isolation effect of natural habitats separating rodents from humans. However, once the percentage exceeded 10%, the risk increased as urbanization created new rodent habitats such as drainage systems and dump sites [16], [42], [43]. The impact of NDVI on HFRS risk exhibited regional heterogeneity. In Northwest China, a U-shaped risk curve was observed, indicating that both sparse vegetation and densely vegetated areas amplify HFRS risk [28], [44]. The findings indicate that various environmental factors interact to collectively shape the ecological niche of HFRS [3], and under such conditions, human activities and increased exposure further contribute to a higher HFRS risk.

For effective HFRS prevention across China, regionally tailored strategies are essential. In Northeast and Northwest China, *Apodemus*-Type epidemic regions, priority should be given to rodent monitoring and control in rural farmland areas. Vaccination of high-risk populations, such as farmers, should be completed prior to the autumn season. Concurrently, the impact of cold weather on HFRS risk should be closely monitored in *Apodemus*-Type epidemic regions. In Central South China, *Rattus*-Type epidemic regions, it is essential to prioritize the management of house mice in residential areas during the spring season, improve indoor sanitation, and implement rodent-controlling measures. In Mixed-Type epidemic regions, a dual-season control strategy is required—implementing rural farmland sanitation campaigns targeting field mice in autumn and winter, while enforcing urban food and waste management measures aimed at house mice in spring.

Several limitations should be acknowledged in this study. First, patients with mild symptoms may not seek medical services and thus might not be captured by the HFRS surveillance system. However, since HFRS reporting is mandatory in China, we consider such underreporting to be minimal. Second, as an ecological study, the analysis did not consider socioeconomic factors, such as GDP and healthcare access, and this study cannot establish causal relationships between meteorological and environmental factors and HFRS incidence. Third, the lack of host surveillance data limited our ability to examine the “Environment-Host-Population” transmission chain. Future research should integrate rodent ecological tracking data to further explore the biological mechanisms underlying HFRS transmission.

## 5 Conclusion

HFRS incidence in China exhibited distinct geographical clustering, with cases concentrated in Northeast, Northwest, and East China, and bimodal seasonality featuring a major peak in autumn/winter and a second peak in spring. The epidemic patterns demonstrated significant regional heterogeneity, mainly due to the differences in host species distribution, meteorological sensitivities, and environmental conditions. Meteorological factors exerted nonlinear lagged effects and threshold effects on HFRS risk, and environmental factors impacted HFRS risk by shaping host ecological niches. Regionally tailored interventions are imperative for HFRS control: targeted vaccination and rodent control in farmlands during autumn for *Apodemus*-Type regions; residential sanitation and house mouse management during spring for *Rattus*-Type regions; and dual-season interventions in both rural and urban settings for Mixed-Type regions.

## CRediT authorship contribution statement

**Chuanlong Cheng:** Writing – review & editing, Writing – original draft, Visualization, Validation, Methodology, Investigation, Formal analysis, Conceptualization. **Michael Tong:** Writing – review & editing, Validation, Supervision, Methodology, Investigation. **Yuchen Hu:** Writing – review & editing, Validation, Supervision, Methodology, Investigation, Formal analysis. **Qi Gao:** Writing – review & editing, Visualization, Validation, Methodology, Investigation, Formal analysis, Data curation. **Hui Zuo:** Writing – review & editing, Visualization, Validation, Methodology, Investigation, Formal analysis, Data curation. **Yuqi Zhang:** Writing – review & editing, Visualization, Validation, Methodology, Investigation, Formal analysis, Data curation. **Liang Lu:** Writing – review & editing, Visualization, Validation, Supervision, Methodology, Investigation, Formal analysis, Data curation, Conceptualization. **Xiujun Li:** Writing – review & editing, Validation, Supervision, Project administration, Funding acquisition, Formal analysis, Data curation, Conceptualization.

## Ethics approval

Not applicable. The data used in this study were case surveillance data, no studies on human subjects were conducted. The ethics approval is not needed.

## Funding

This work was supported by 10.13039/501100012166National Key Research and Development Program of China [grant numbers: 2023YFC2604401] and 10.13039/501100001809National Natural Science Foundation of China [grant numbers: 81673238].

## Declaration of competing interest

The authors report there are no competing interests to declare.

## Data Availability

HFRS data supporting the results of this study were available from the China Information System for Disease Control and Prevention (CISDCP), but the availability of these data was limited, which was used under the license of the project we are conducting. If appropriate permission for the data is required, please apply through the project to obtain the license.

## References

[1] Sehgal A., Mehta S., Sahay K. (2023). Hemorrhagic fever with renal syndrome in Asia: History, pathogenesis, diagnosis, treatment, and prevention. Viruses.

[2] Wang Y., Wei X., Xiao X. (2022). Climate and socio-economic factors drive the spatio-temporal dynamics of HFRS in northeastern China. One Health..

[3] Wen B., Yang Z., Ren S. (2024). Spatial-temporal patterns and influencing factors for hemorrhagic fever with renal syndrome: a 16-year national surveillance analysis in China. One Health..

[4] He J., Wang Y., Wei X. (2023). Spatial-temporal dynamics and time series prediction of HFRS in mainland China: A long-term retrospective study. J. Med. Virol..

[5] Wang Y., Zhang C., Gao J. (2024). Spatiotemporal trends of hemorrhagic fever with renal syndrome (HFRS) in China under climate variation. Proc. Natl. Acad. Sci. USA.

[6] Luo Y., Zhang L., Xu Y. (2024). Epidemic characteristics and meteorological risk factors of hemorrhagic fever with renal syndrome in 151 cities in China from 2015 to 2021: retrospective analysis. JMIR Public Health Surveill..

[7] Lv C.L., Tian Y., Qiu Y. (2023). Dual seasonal pattern for hemorrhagic fever with renal syndrome and its potential determinants in China. Sci. Total Environ..

[8] Li Y., Cazelles B., Yang G. (2019). Intrinsic and extrinsic drivers of transmission dynamics of hemorrhagic fever with renal syndrome caused by Seoul hantavirus. PLoS Negl. Trop. Dis..

[9] de Souza W.M., Weaver S.C. (2024). Effects of climate change and human activities on vector-borne diseases. Nat. Rev. Microbiol..

[10] Wang Y.X.G., Voutilainen L., Aminikhah M. (2023). The impact of wildlife and environmental factors on hantavirus infection in the host and its translation into human risk. Proc. R. Soc. B Biol. Sci..

[11] Keesing F., Ostfeld R.S. (2024). Emerging patterns in rodent-borne zoonotic diseases. Science.

[12] Chen L., Liu D., Guo Y. (2025). Impact of climate change and extreme temperature on the incidence of infectious disease among children and adolescents in China: a nationwide case-crossover study with over 8.7 million cases between 2008 and 2019. J. Inf. Secur..

[13] Tian H., Yu P., Bjornstad O.N. (2017). Anthropogenically driven environmental changes shift the ecological dynamics of hemorrhagic fever with renal syndrome. PLoS Pathog..

[14] Ji H., Li K., Shang M. (2024). The 2016 severe floods and incidence of hemorrhagic fever with renal syndrome in the Yangtze River basin. JAMA Netw. Open.

[15] Wang Z., Pei S., Cui H. (2024). Zoonotic spillover and extreme weather events drive the global outbreaks of airborne viral emerging infectious diseases. J. Med. Virol..

[16] Tian H., Hu S., Cazelles B. (2018). Urbanization prolongs hantavirus epidemics in cities. Proc. Natl. Acad. Sci. USA.

[17] Garcia-Pena G.E., Rubio A.V., Mendoza H. (2021). Land-use change and rodent-borne diseases: hazards on the shared socioeconomic pathways. Philosoph. Transact. Royal Soc. B-Biol. Sci..

[18] Guterres A., de Lemos E.R.S. (2018). Hantaviruses and a neglected environmental determinant. One Health..

[19] Chen J.J., Guo T.C., Song S.X. (2020). Epidemiological characteristics and the development of spatiotemporal analysis models on hemorrhagic fever with renal syndrome in China. Zhonghua Liu Xing Bing Xue Za Zhi.

[20] ECMWF (2019). ERA5-Land Monthly Averaged Data from 1950 to Present. https://www.ecmwf.int/en/forecasts/dataset/ecmwf-reanalysis-v5.

[21] Jixi G., Yuanli S., Hongwei Z. (2022).

[22] Guoan T. (2019). https://data.tpdc.ac.cn/zh-hans/data/12e91073-0181-44bf-8308-c50e5bd9a734.

[23] Gao Jixi, Yuanli S., Hongwei Z. (2022).

[24] Yang J., Huang X. (2024). Earth System Science Data.

[25] Gasparrini A., Armstrong B., Kenward M.G. (2012). Multivariate meta-analysis for non-linear and other multi-parameter associations. Stat. Med..

[26] Chen C. (2000). Generalized additive mixed models. Commun. Stat. Theory Meth..

[27] R Core Team (2025). https://www.R-project.org/.

[28] Zhu L., Lu L., Li S. (2023). Spatiotemporal variations and potential influencing factors of hemorrhagic fever with renal syndrome: a case study in Weihe Basin, China. PLoS Negl. Trop. Dis..

[29] Xue C., Zhang B., Li Y. (2024). Asymmetric association between meteorological factors and human infections with hemorrhagic fever with renal syndrome: a 16-year ecological trend study in Shaanxi, China. One Health.

[30] Chang N., Huang W., Niu Y. (2025). Risk of hemorrhagic fever with renal syndrome associated with meteorological factors in diverse epidemic regions: a nationwide longitudinal study in China. Infect. Dis. Poverty.

[31] Liang W., Gu X., Li X. (2018). Mapping the epidemic changes and risks of hemorrhagic fever with renal syndrome in Shaanxi Province, China, 2005–2016. Sci. Rep..

[32] Li G., Wan X., Yin B. (2021). Timing outweighs magnitude of rainfall in shaping population dynamics of a small mammal species in steppe grassland. Proc. Natl. Acad. Sci. USA.

[33] Xiang J., Hansen A., Liu Q. (2018). Impact of meteorological factors on hemorrhagic fever with renal syndrome in 19 cities in China, 2005-2014. Sci. Total Environ..

[34] Hansen A., Cameron S., Liu Q. (2015). Transmission of haemorrhagic fever with renal syndrome in China and the role of climate factors: a review. Int. J. Infect. Dis..

[35] Bai X.H., Peng C., Jiang T. (2019). Distribution of geographical scale, data aggregation unit and period in the correlation analysis between temperature and incidence of HFRS in mainland China: a systematic review of 27 ecological studies. PLoS Negl. Trop. Dis..

[36] Lin H., Zhang Z., Lu L. (2014). Meteorological factors are associated with hemorrhagic fever with renal syndrome in Jiaonan County, China, 2006-2011. Int. J. Biometeorol..

[37] Zhang W.-Y., Guo W.-D., Fang L.-Q. (2010). Climate variability and hemorrhagic fever with renal syndrome transmission in northeastern China. Environ. Health Perspect..

[38] Xiao H., Tian H.-Y., Gao L.-D. (2014). Animal reservoir, natural and socioeconomic variations and the transmission of hemorrhagic fever with renal syndrome in Chenzhou, China, 2006–2010. PLoS Negl. Trop. Dis..

[39] Yan L., Fang L.Q., Huang H.G. (2007). Landscape elements and Hantaan virus-related hemorrhagic fever with renal syndrome, People’s Republic of China. Emerg. Infect. Dis..

[40] Xiao H., Tong X., Gao L. (2018). Spatial heterogeneity of hemorrhagic fever with renal syndrome is driven by environmental factors and rodent community composition. PLoS Negl. Trop. Dis..

[41] Hassell J.M., Newbold T., Dobson A.P. (2021). Towards an ecosystem model of infectious disease. Nat Ecol Evol..

[42] Kim D. (2021). Exploratory study on the spatial relationship between emerging infectious diseases and urban characteristics: cases from Korea. Sustain. Cities Soc..

[43] Shen L., Sun M., Wei X. (2022). Spatiotemporal association of rapid urbanization and water-body distribution on hemorrhagic fever with renal syndrome: a case study in the city of Xi’an, China. PLoS Negl. Trop. Dis..

[44] Zheng L., Gao Q., Yu S. (2024). Using empirical dynamic modeling to identify the impact of meteorological factors on hemorrhagic fever with renal syndrome in Weifang, northeastern China, from 2011 to 2020. PLoS Negl. Trop. Dis..

