# Supplementary material for: Regional heterogeneity of spatial-temporal characteristics and meteorological-environmental driving factors of hemorrhagic fever with renal syndrome in China
Source: One Health. 2026 May 20;22:101454. doi: 10.1016/j.onehlt.2026.101454 (PMC13223968; doi:10.1016/j.onehlt.2026.101454)
Supplement: Supplementary file 1 — Supplementary material [file mmc1.pdf]

Regional Heterogeneity of Spatial-Temporal Characteristics and Meteorological-Environmental Driving Factors of Hemorrhagic Fever with Renal Syndrome in China

Chuanlong Cheng [a], Michael Tong [b], Yuchen Hu [c], Qi Gao [a], Hui Zuo [a], Yuqi Zhang [a], Liang Lu [d], Xiujun Li [a,*]

a Department of Biostatistics, School of Public Health, Cheeloo College of Medicine, Shandong University, Jinan, 250012, Shandong, China.

b National Centre for Epidemiology and Population Health, The Australian National University, Canberra, Australian Capital Territory 2601, Australia.

c Institute of Clinical Trials and Methodology, MRC Clinical Trials Unit, University College London, London, WC1V 6LJ, UK.

d National Key Laboratory of Intelligent Tracking and Forecasting for Infectious Diseases, National Institute for Communicable Disease Control and Prevention, Chinese Center for Disease Control and Prevention, Beijing, 102211, China.

*Correspondence to:

Xiujun Li,

Email: xjli@sdu.edu.cn;

44# Wenhuaxi Road, Lixia District, Jinan, Shandong 250012, People's Republic of China

Contents

Methods 3
Table S1. Summary of the study cities. 5
Table S2. Description of environmental factors. 8
Table S3. Incidence of HFRS in provinces with high incidence in China. 10
Figure S1. Monthly trend of incidence of HFRS in provinces with high incidence of HFRS in China. 10
Figure S2. Seasonal trends in the incidence of HFRS in different epidemic type regions. 11
Figure S3. Seasonal trends in the incidence of HFRS in different climate zones. 11
Figure S4. Seasonal trends in the incidence of HFRS in different geographical regions. 12
Figure S5. Composition of epidemic types in HFRS in different climate zones. 13
Figure S6. Composition of epidemic types in HFRS in different geographical regions. 13
Figure S7. Lag effects of meteorological factors on the incidence of HFRS. 14
Figure S8. Nonlinear effects of meteorological factors in regions with different epidemic types. 15
Figure S9. Nonlinear effects of meteorological factors in different climate zones. 16
Figure S10. Nonlinear effects of meteorological factors in different geographical regions. 17
Figure S11. Differences in effects of relative humidity, precipitation and snow depth in different regions. 18
Figure S12. Correlations of environmental factors. 19
Figure S13. Relative importance of environmental factors. 19
Figure S14. Correlation and importance of environmental factors in *Apodemus*-Type regions. 20
Figure S15. Correlation and importance of environmental factors in *Rattus*-Type regions. 20
Figure S16. Correlation and importance of environmental factors in Mixed-Type regions. 20
Figure S17. Effects of environmental factors in *Apodemus*-Type regions. 21
Figure S18. Effects of environmental factors in *Rattus*-Type regions. 22
Figure S19. Effects of environmental factors in Mixed-Type regions. 23
Figure S20. Correlation and importance of environmental factors in mid-temperate zone. 24
Figure S21. Correlation and importance of environmental factors in warm temperate zone. 24
Figure S22. Correlation and importance of environmental factors in subtropical zone. 24
Figure S23. Differences in the effects of environmental factors in different climate zones. 25
Figure S24. Effects of environmental factors in mid-temperate zone. 26
Figure S25. Effects of environmental factors in warm temperate zone. 27
Figure S26. Effects of environmental factors in subtropical zone. 28
Figure S27. Correlation and importance of environmental factors in Northeast China. 29
Figure S28. Correlation and importance of environmental factors in East China. 29
Figure S29. Correlation and importance of environmental factors in Northwest China. 30
Figure S30. Correlation and importance of environmental factors in Central South China. 30
Figure S31. Differences in the effects of environmental factors in different geographical regions. 31
Figure S32. Effect of environmental factors in Northeast China. 32
Figure S33. Effects of environmental factors in East China. 33
Figure S34. Effects of environmental factors in Northwest China. 34
Figure S35. Effects of environmental factors in Central South China. 35
Figure S36. Sensitivity analysis of DLNM. 36
Figure S37. Sensitivity analysis of GAMM. 37
References 38

Methods

Analysis of Meteorological Factor Impacts using a Two-Stage Strategy

The specific DLNM used in this study is:

$$log[E(Y_{t,k})] = \alpha_k + cb(Var_{t,l,k};\theta_{l,k}) + \sum_{i=1}^{N} ns(X_{i,l,k}, df; \beta_{i,l,k}) + ns(Time, df) + log(POP_{t,k})$$

Where， $Y_{t,k}$ is the number of HFRS cases in city k during month t; α_k is the intercept for city k; cb represents the cross-basis function， $Var_{t,l,k}$ is a specific meteorological variable, where l is the lag time (determined as 6 months based on previous studies and model fit), using natural splines with 3 degrees of freedom (*df*) each for the non-linear effect of the variable and the lag dimension, and $\theta_{l,k}$ are the fitted coefficients; *ns* represents natural spline function used to model non−linear effects; $X_{i,l,k}$ represents other meteorological variables with cumulative lag l (consistent with the cross−basis lag), modeled using natural splines with 3 df each; $Time$ is the monthly time variable during the study period, controlling for long−term and seasonal trends, modeled with a natural spline (*df*=18); $POP_{t,k}$ is the population of city k in month t. All dfs settings were selected based on minimizing the Quasi-Akaike Information Criterion (QAIC). To avoid strong correlations between different meteorological factors, control variables were selectively included when investigating the effect of a single factor.

According to the purpose of the study and the correlation of variables, the model variable combinations in this study are the following six:

[1] Monthly mean temperature + (monthly relative humidity, precipitation) + others

[2] Monthly maximum temperature + (monthly relative humidity, precipitation) + others

[3] Monthly minimum temperature + (monthly relative humidity, precipitation) + others

[4] Monthly relative humidity + (monthly mean temperature, precipitation) + others

[5] Monthly precipitation + (monthly mean temperature, relative humidity) + others

[6] Monthly snow depth + (monthly mean temperature, relative humidity, precipitation) + others

Analysis of Environmental Factor Impacts using Generalized Additive Mixed Models

The specific GAMM form is:

$$log[E(Y_{t,k})] = \alpha + b_k + \sum_{i=1}^{N} s_i(X_{i,t,k}) + s(year) + log(POP_{t,k}) + \varepsilon_{t,k}$$

Where $Y_{t,k}$ is the number of HFRS cases in city k during year t; α is the intercept; b_k is the random effect for city k; s_i represents cubic spline functions for the *i*-th environmental factor $X_{i,t,k}$; $year$ is the

time variable, controlled using a cubic spline function to account for temporal trends; $POP_{t,k}$ is the population of city k in month t; $\varepsilon_{t,k}$ is the model residual. Quasi-Poisson regression was used to analyze the relationship between influencing factors and HFRS epidemics, suitable for count data and capturing non-linear features. Spline functions were used for modeling to accommodate data complexity. City-level random effects were introduced to account for heterogeneity among cities, reducing bias.

According to the seasonal distribution characteristics of the cases, the study cities were divided into three types of epidemic regions. Many previous studies showed that HFRS cases mainly occurred during autumn and winter in the *Apodemus*-Type epidemic regions, during spring in the *Rattus*-Type epidemic regions, and cases were reported in both periods in the Mixed-Type epidemic regions. Firstly, according to studies on the distribution of hantavirus serotypes and host animal surveillance at home and abroad, we identified the types of cities that have already been classified. Furthermore, according to the seasonal distribution of HFRS cases, we identified types of the cities that have not been classified. The accuracy of the city type classification in this paper was checked by referring to the previous research results[1,2]. In addition, according to the Chinese authorities released the climate zone division (https://www.resdc.cn/data.aspx?DATAID=243) and the geographic map data (https://www.resdc.cn/data.aspx?DATAID=276), based on the geographic coordinates of the center of each city, the corresponding regions was matched, and the types of climate zone and geographical region for each city were finally determined. The city-type classification work described above was carried out simultaneously by CCL and GQ, and multiple checks were performed to ensure the accuracy of the classification.

Table S1. Summary of the study cities.

City	IDCity	Province	Cases	Type of Epidemic	Climate Zone	Geographical Region
Harbin	230100	Heilongjiang	4170	Mixed	Mid-Temperate Zone	Northeast China
Qiqihar	230200	Heilongjiang	3337	Apodemus	Mid-Temperate Zone	Northeast China
Jixi	230300	Heilongjiang	2614	Apodemus	Mid-Temperate Zone	Northeast China
Hegang	230400	Heilongjiang	1562	Apodemus	Mid-Temperate Zone	Northeast China
Shuangyashan	230500	Heilongjiang	3594	Mixed	Mid-Temperate Zone	Northeast China
Daqing	230600	Heilongjiang	645	Rattus	Mid-Temperate Zone	Northeast China
Yichun (HLJ)	230700	Heilongjiang	834	Apodemus	Mid-Temperate Zone	Northeast China
Jiamusi	230800	Heilongjiang	5314	Apodemus	Mid-Temperate Zone	Northeast China
Qitaihe	230900	Heilongjiang	483	Apodemus	Mid-Temperate Zone	Northeast China
Mudanjiang	231000	Heilongjiang	3671	Apodemus	Mid-Temperate Zone	Northeast China
Heihe	231100	Heilongjiang	3112	Apodemus	Mid-Temperate Zone	Northeast China
Suihua	231200	Heilongjiang	2309	Mixed	Mid-Temperate Zone	Northeast China
Xi'an	610100	Shaanxi	15809	Apodemus	WarmTemperate Zone	Northwest China
Tongchuan	610200	Shaanxi	377	Apodemus	WarmTemperate Zone	Northwest China
Baoji	610300	Shaanxi	3983	Mixed	WarmTemperate Zone	Northwest China
Xianyang	610400	Shaanxi	5322	Apodemus	WarmTemperate Zone	Northwest China
Weinan	610500	Shaanxi	4958	Apodemus	WarmTemperate Zone	Northwest China
Yan'an	610600	Shaanxi	399	Apodemus	WarmTemperate Zone	Northwest China
Ankang	610900	Shaanxi	121	Apodemus	WarmTemperate Zone	Northwest China
Shangluo	611000	Shaanxi	441	Apodemus	WarmTemperate Zone	Northwest China
Changchun	220100	Jilin	2597	Rattus	Mid-Temperate Zone	Northeast China
Jilin	220200	Jilin	1934	Mixed	Mid-Temperate Zone	Northeast China
Siping	220300	Jilin	1316	Rattus	Mid-Temperate Zone	Northeast China
Liaoyuan	220400	Jilin	868	Rattus	Mid-Temperate Zone	Northeast China
Tonghua	220500	Jilin	2240	Mixed	Mid-Temperate Zone	Northeast China
Baishan	220600	Jilin	1462	Mixed	Mid-Temperate Zone	Northeast China
Songyuan	220700	Jilin	469	Rattus	Mid-Temperate Zone	Northeast China
Baicheng	220800	Jilin	1102	Rattus	Mid-Temperate Zone	Northeast China
Yanbian	222400	Jilin	2653	Mixed	Mid-Temperate Zone	Northeast China
Shenyang	210100	Liaoning	1915	Mixed	Mid-Temperate Zone	Northeast China
Dalian	210200	Liaoning	722	Mixed	Mid-Temperate Zone	Northeast China
Anshan	210300	Liaoning	1226	Mixed	Mid-Temperate Zone	Northeast China
Fushun	210400	Liaoning	2324	Apodemus	Mid-Temperate Zone	Northeast China
Benxi	210500	Liaoning	1450	Mixed	Mid-Temperate Zone	Northeast China
Dandong	210600	Liaoning	1755	Apodemus	Mid-Temperate Zone	Northeast China
Jinzhou	210700	Liaoning	2820	Rattus	Mid-Temperate Zone	Northeast China
Yingkou	210800	Liaoning	1028	Rattus	Mid-Temperate Zone	Northeast China
Liaoyang	211000	Liaoning	212	Apodemus	Mid-Temperate Zone	Northeast China
Panjin	211100	Liaoning	328	Rattus	Mid-Temperate Zone	Northeast China
Tieling	211200	Liaoning	1719	Mixed	Mid-Temperate Zone	Northeast China
Chaoyang	211300	Liaoning	1057	Rattus	Mid-Temperate Zone	Northeast China
Huludao	211400	Liaoning	4094	Rattus	Mid-Temperate Zone	Northeast China

City	IDCity	Province	Cases	Type of Epidemic	Climate Zone	Geographical Region
Jinan	370100	Shandong	1511	Apodemus	WarmTemperate Zone	East China
Qingdao	370200	Shandong	3066	Apodemus	WarmTemperate Zone	East China
Zibo	370300	Shandong	1677	Mixed	WarmTemperate Zone	East China
Zaozhuang	370400	Shandong	432	Rattus	WarmTemperate Zone	East China
Dongying	370500	Shandong	285	Mixed	WarmTemperate Zone	East China
Yantai	370600	Shandong	1600	Apodemus	WarmTemperate Zone	East China
Weifang	370700	Shandong	3898	Apodemus	WarmTemperate Zone	East China
Jining	370800	Shandong	1320	Mixed	WarmTemperate Zone	East China
Tai'an	370900	Shandong	503	Rattus	WarmTemperate Zone	East China
Weihai	371000	Shandong	552	Mixed	WarmTemperate Zone	East China
Rizhao	371100	Shandong	2304	Apodemus	WarmTemperate Zone	East China
Linyi	371300	Shandong	2877	Mixed	WarmTemperate Zone	East China
Dezhou	371400	Shandong	121	Rattus	WarmTemperate Zone	East China
Binzhou	371600	Shandong	667	Mixed	WarmTemperate Zone	East China
Heze	371700	Shandong	330	Mixed	WarmTemperate Zone	East China
Nanchang	360100	Jiangxi	846	Rattus	Subtropical Zone	East China
Jiujiang	360400	Jiangxi	171	Rattus	Subtropical Zone	East China
Xinyu	360500	Jiangxi	271	Mixed	Subtropical Zone	East China
Yingtan	360600	Jiangxi	173	Mixed	Subtropical Zone	East China
Ji'an	360800	Jiangxi	388	Apodemus	Subtropical Zone	East China
Yichun (JX)	360900	Jiangxi	4492	Mixed	Subtropical Zone	East China
Fuzhou(JX)	361000	Jiangxi	737	Apodemus	Subtropical Zone	East China
Shangrao	361100	Jiangxi	2064	Mixed	Subtropical Zone	East China
Changsha	430100	Hunan	1964	Mixed	Subtropical Zone	Central South China
Zhuzhou	430200	Hunan	285	Apodemus	Subtropical Zone	Central South China
Xiangtan	430300	Hunan	908	Mixed	Subtropical Zone	Central South China
Hengyang	430400	Hunan	829	Mixed	Subtropical Zone	Central South China
Shaoyang	430500	Hunan	1487	Mixed	Subtropical Zone	Central South China
Yueyang	430600	Hunan	449	Mixed	Subtropical Zone	Central South China
Changde	430700	Hunan	724	Rattus	Subtropical Zone	Central South China
Yiyang	430900	Hunan	751	Mixed	Subtropical Zone	Central South China
Chenzhou	431000	Hunan	1108	Mixed	Subtropical Zone	Central South China
Yongzhou	431100	Hunan	521	Mixed	Subtropical Zone	Central South China
Huaihua	431200	Hunan	727	Apodemus	Subtropical Zone	Central South China
Loudi	431300	Hunan	1431	Mixed	Subtropical Zone	Central South China
Shijiazhuang	130100	Hebei	604	Rattus	WarmTemperate Zone	North China
Tangshan	130200	Hebei	3372	Mixed	WarmTemperate Zone	North China
Qinhuangdao	130300	Hebei	4632	Rattus	WarmTemperate Zone	North China
Handan	130400	Hebei	204	Apodemus	WarmTemperate Zone	North China
Xingtai	130500	Hebei	219	Rattus	WarmTemperate Zone	North China
Baoding	130600	Hebei	451	Mixed	WarmTemperate Zone	North China
Zhangjiakou	130700	Hebei	292	Rattus	WarmTemperate Zone	North China
Chengde	130800	Hebei	472	Rattus	WarmTemperate Zone	North China
Cangzhou	130900	Hebei	284	Mixed	WarmTemperate Zone	North China

City	IDCity	Province	Cases	Type of Epidemic	Climate Zone	Geographical Region
Hengshui	131100	Hebei	108	Rattus	WarmTemperate Zone	North China
Fuzhou(FJ)	350100	Fujian	840	Mixed	Subtropical Zone	East China
Xiamen	350200	Fujian	102	Rattus	Subtropical Zone	East China
Putian	350300	Fujian	238	Mixed	Subtropical Zone	East China
Sanming	350400	Fujian	590	Rattus	Subtropical Zone	East China
Quanzhou	350500	Fujian	885	Mixed	Subtropical Zone	East China
Zhangzhou	350600	Fujian	370	Rattus	Subtropical Zone	East China
Nanping	350700	Fujian	1787	Mixed	Subtropical Zone	East China
Ningde	350900	Fujian	786	Mixed	Subtropical Zone	East China
Hangzhou	330100	Zhejiang	388	Mixed	Subtropical Zone	East China
Ningpo	330200	Zhejiang	1959	Mixed	Subtropical Zone	East China
Wenzhou	330300	Zhejiang	256	Mixed	Subtropical Zone	East China
Huzhou	330500	Zhejiang	187	Rattus	Subtropical Zone	East China
Shaoxing	330600	Zhejiang	1177	Mixed	Subtropical Zone	East China
Jinhua	330700	Zhejiang	678	Mixed	Subtropical Zone	East China
Quzhou	330800	Zhejiang	866	Mixed	Subtropical Zone	East China
Taizhou	331000	Zhejiang	1702	Mixed	Subtropical Zone	East China
Lishui	331100	Zhejiang	916	Mixed	Subtropical Zone	East China
Wuhan	420100	Hubei	137	Mixed	Subtropical Zone	Central South China
Huangshi	420200	Hubei	186	Mixed	Subtropical Zone	Central South China
Yichang	420500	Hubei	119	Mixed	Subtropical Zone	Central South China
Xiangyang	420600	Hubei	1170	Mixed	Subtropical Zone	Central South China
Jingmen	420800	Hubei	750	Mixed	Subtropical Zone	Central South China
Xiaogan	420900	Hubei	163	Mixed	Subtropical Zone	Central South China
Jingzhou	421000	Hubei	1492	Rattus	Subtropical Zone	Central South China
Huanggang	421100	Hubei	250	Rattus	Subtropical Zone	Central South China
Xianning	421200	Hubei	131	Mixed	Subtropical Zone	Central South China
Xiantao	429004	Hubei	323	Mixed	Subtropical Zone	Central South China
Qianjiang	429005	Hubei	816	Rattus	Subtropical Zone	Central South China
Tianmen	429006	Hubei	623	Mixed	Subtropical Zone	Central South China

Table S2. Description of environmental factors.

Environmental Factors	Variable	Description	Data Sources
Vegetational Cover	NDVI[3]	Normalized Difference Vegetation Index (NDVI) is a remote sensing index used to assess vegetation cover and health. It reflects the density, growth state and biomass of vegetation by calculating the reflectance difference between Red (Red) and near-infrared (NIR) bands. It ranges from -1 to 1, and the higher the value, the more dense the vegetation.。	https://data.tpdc.ac.cn/home
	Vegetation Coverage (%)[4]	Vegetation Coverage refers to the proportion of land surface area covered by vegetation (such as forest, grassland, farmland, etc.) in a certain area, which is an important indicator to measure ecosystem health and surface environmental quality.	
Land Use[5]	Cropland (%)	The arable land and human planted crops not at tree height including upland crops such as wheat, corn, potatoes, and cotton, and irrigated crops such as paddy filed, lotus root, and water spinach.	https://zenodo.org/records/12779975
	Forest (%)	Areas covered by trees generally have larger crowns and are higher than 5 meters. It can be sparse arbors or clustered forests which include evergreen forests, mixed forests, artificial forests, bamboo groves, etc.	
	Shrub (%)	Areas covered by clusters of shrubs with a height below 5 meters.	
	Grassland (%)	Areas covered by low herbaceous plants. It generally includes natural grasslands with a fractional vegetation coverage greater than 5, rangeland with tree canopy density less than 0.3 or shrub canopy density less than 0.4, urban's vacant land dominated by grass, and other artificial grasslands.	
	Water (%)	Areas covered by water for a long period, including oceans, naturally formed water bodies such as lakes, rivers, and runoff, artificially formed water bodies such as reservoirs, canals, water conservancy facilities (with open water), ponds, and aquaculture farms.	
	Snow/Ice (%)	Areas covered by large-scale permanent snow or ice, including glaciers and permanent snowpack in mountain areas or high latitudes.	

	Barren (%)	Areas covered by sparse vegetation or bare land covered by sand, gravel, or rocks, including mountains without dense vegetation and snow cover, deserts, grasslands degraded by drought, and wasteland in urban/rural areas with sparse or no vegetation.	
	Impervious (%)	Human-made structures and homogenous impervious surfaces including industrial, residential, commercial areas, construction sites, railways, highways, etc. It is generally located in urban and rural areas with high human activities.	
	Wetland (%)	Areas with perennial or seasonal water accumulation and vegetation growth. It includes forest/shrub/grass swamps, peatlands, mudflat, mangroves, and coastal/inland tidal flats.	
DEM[6]	Elevation (m)	Elevation refers to the vertical distance between a point on the ground and the sea level, that is, the height difference, usually calculated based on the average sea level.	https://data.tpdc.ac.cn/home
	Slope (°)	Slope, also known as slope ratio, is the ratio of the vertical height h of the slope and the horizontal width l of the slope, which is the tangent value of the slope Angle.	
	Slope Orientation (°)	The slope orientation is defined as the direction of the projection of the slope normal on the horizontal plane, that is, the direction from high to low. Slope orientation plays an important role in mountain ecology. The position of the mountain affected the sunshine duration and solar radiation intensity. For the northern hemisphere, the south slope had the most radiation income, followed by the southeast and southwest slopes, then the east and west slopes, the northeast and northwest slopes, and the north slope had the least radiation income.	
River[7]	River Distance (m)	The dataset includes all river networks in the country and all subbasins with an area greater than 100 km2. The distance between the central point of the city and the river was calculated.	https://www.resdc.cn

Table S3. Incidence of HFRS in provinces with high incidence in China.

Geographical Region	Province	Population (100,000)	Cumulative Cases	Cumulative Incidence (1/100,000)	Annual Incidence (1/100,000)
Northeast China	Heilongjiang	318.501	31,805	99.858	5.548
Northwest China	Shaanxi	395.290	31,544	79.800	4.433
Northeast China	Jilin	240.735	14,641	60.818	3.379
Northeast China	Liaoning	425.914	20,698	48.597	2.700
East China	Shandong	1,015.275	21,233	20.914	1.162
East China	Jiangxi	451.886	9,246	20.461	1.137
Central South China	Hunan	664.449	11,291	16.993	0.944
North China	Hebei	746.102	10,684	14.320	0.796
East China	Fujian	415.401	5,624	13.539	0.752
East China	Zhejiang	645.676	8,171	12.655	0.703
Central South China	Hubei	577.526	6,295	10.900	0.606

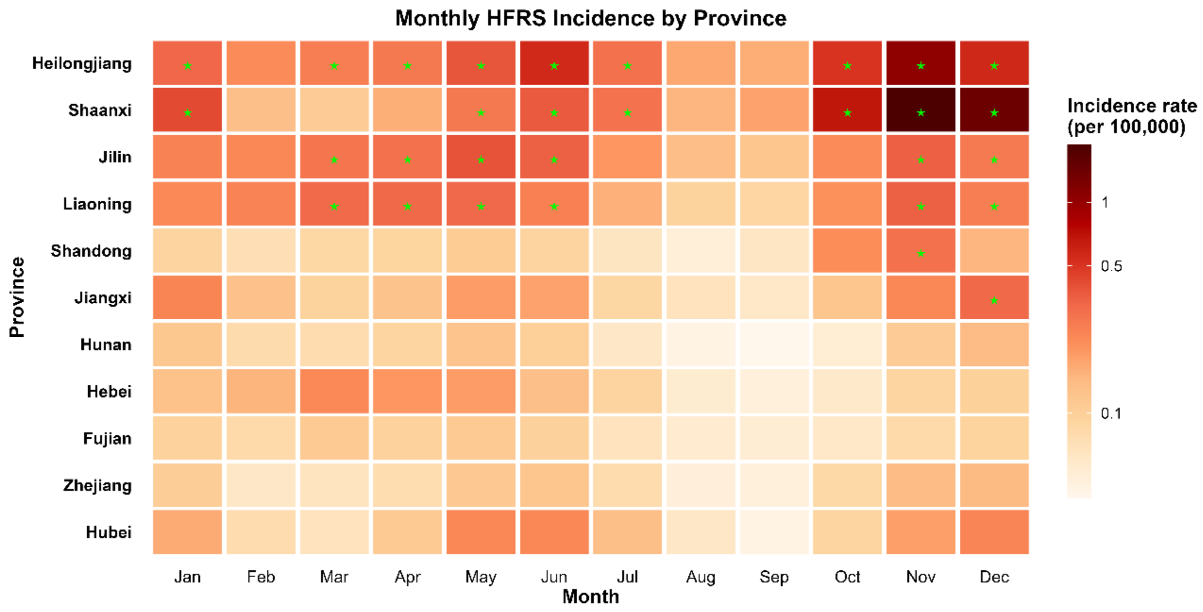

Figure S1. Monthly trend of incidence of HFRS in provinces with high incidence of HFRS in China. (Note: asterisk represents monthly incidence greater than the national average level)

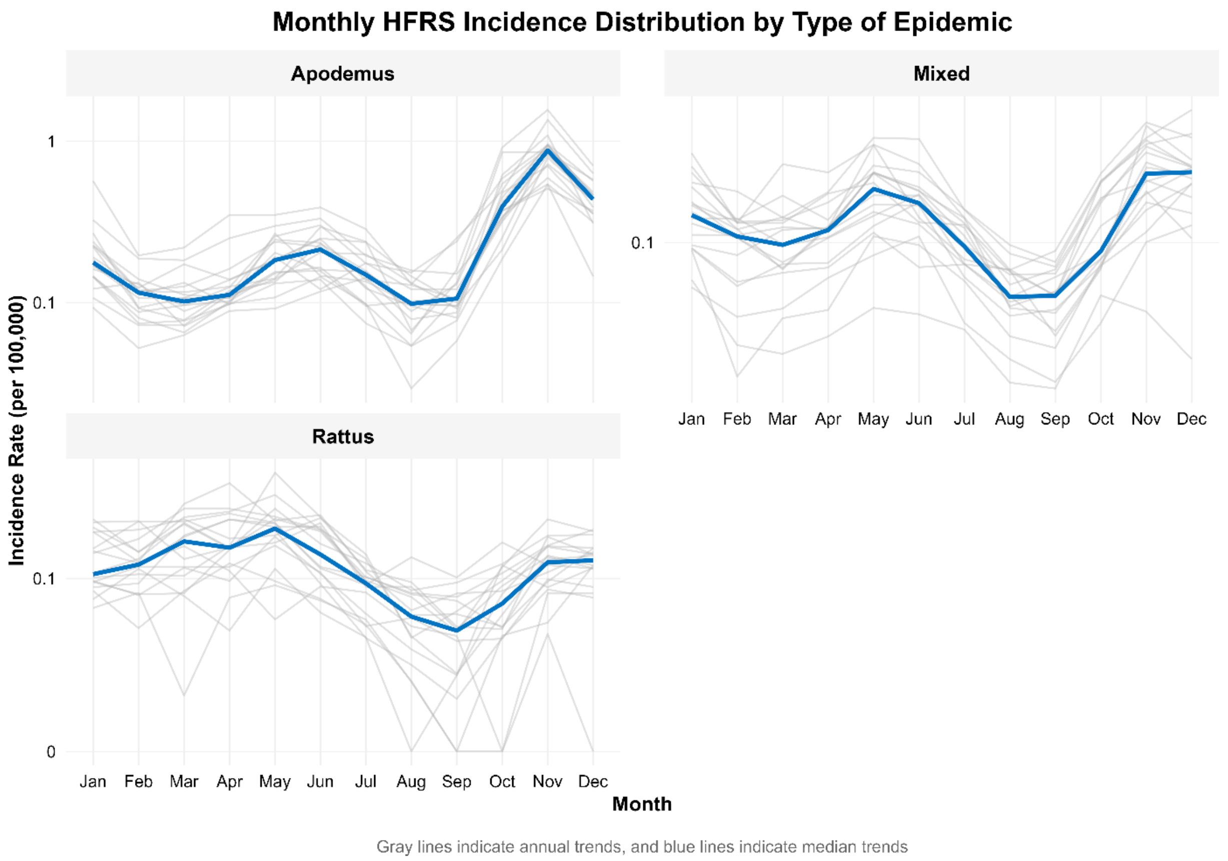

Figure S2. Seasonal trends in the incidence of HFRS in different epidemic type regions.

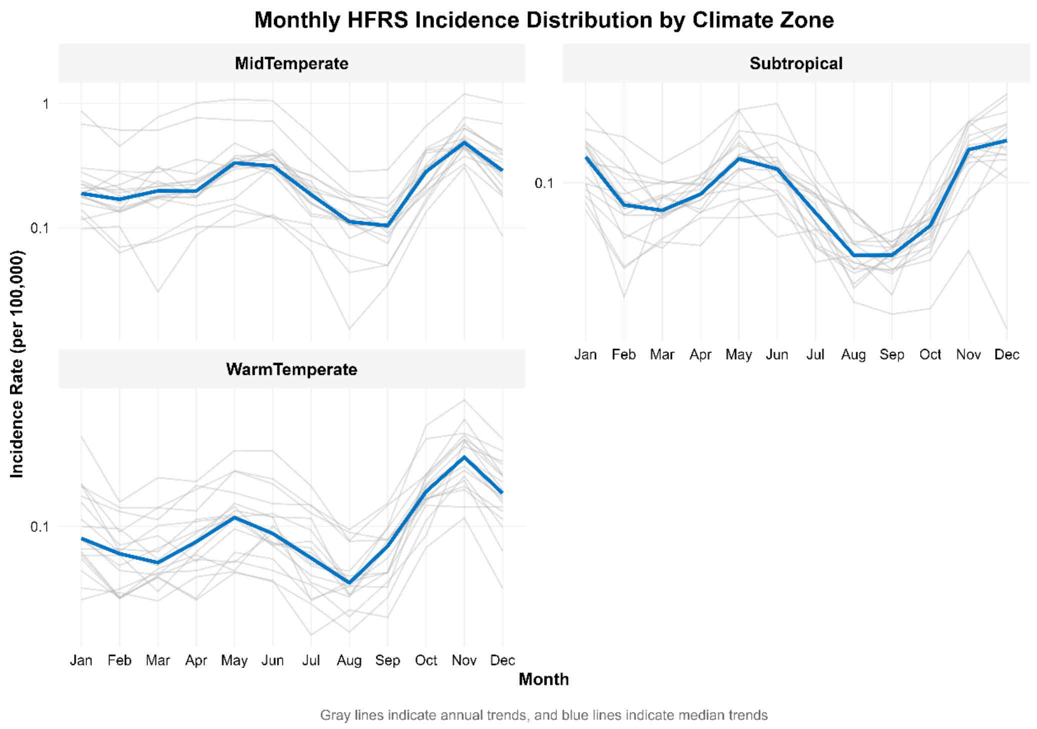

Figure S3. Seasonal trends in the incidence of HFRS in different climate zones.

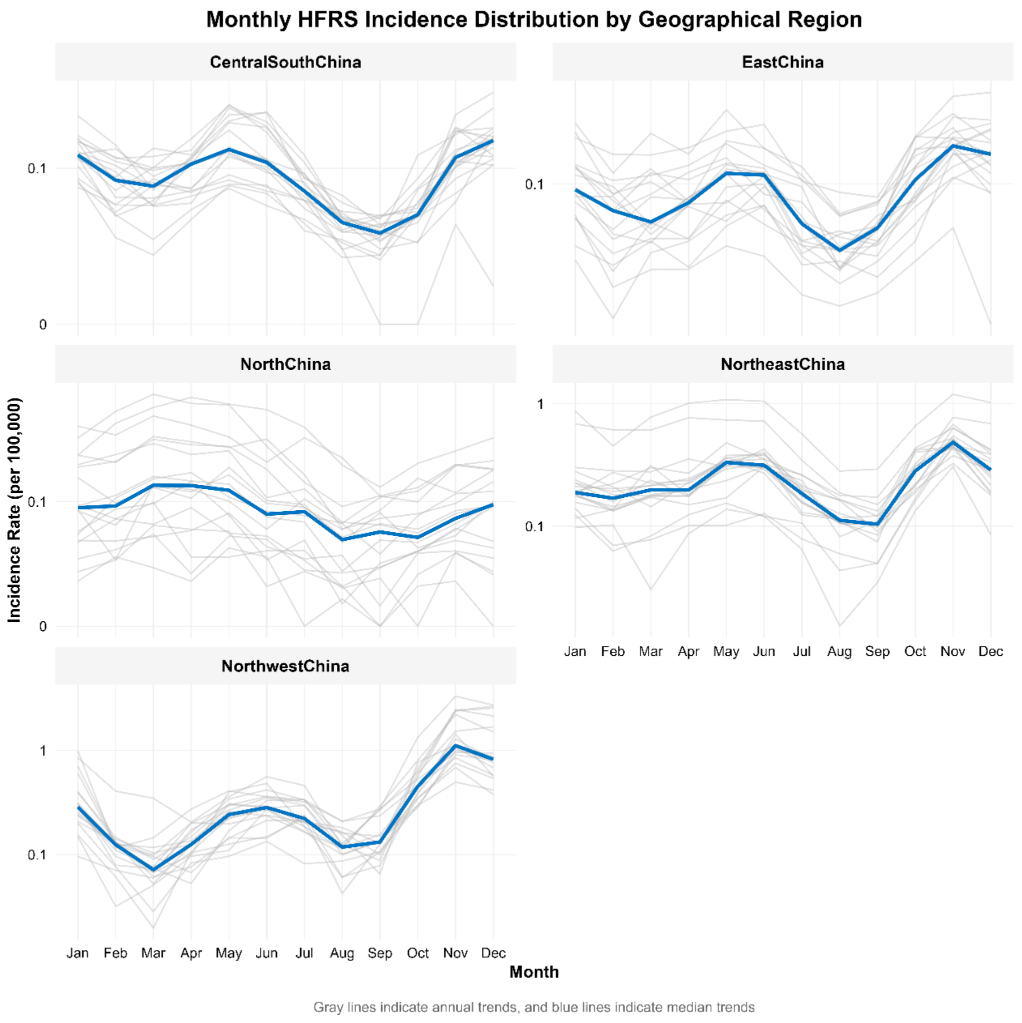

Figure S4. Seasonal trends in the incidence of HFRS in different geographical regions.

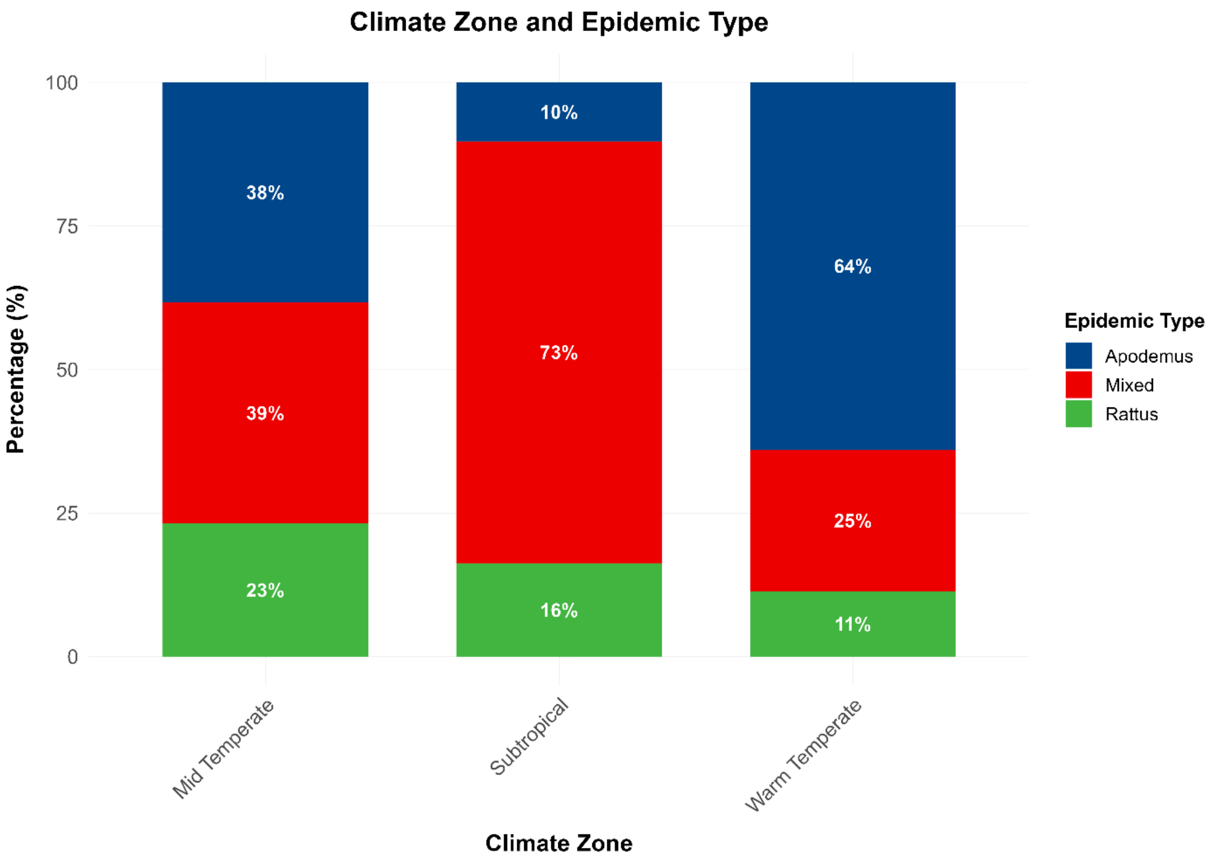

Figure S5. Composition of epidemic types in HFRS in different climate zones.

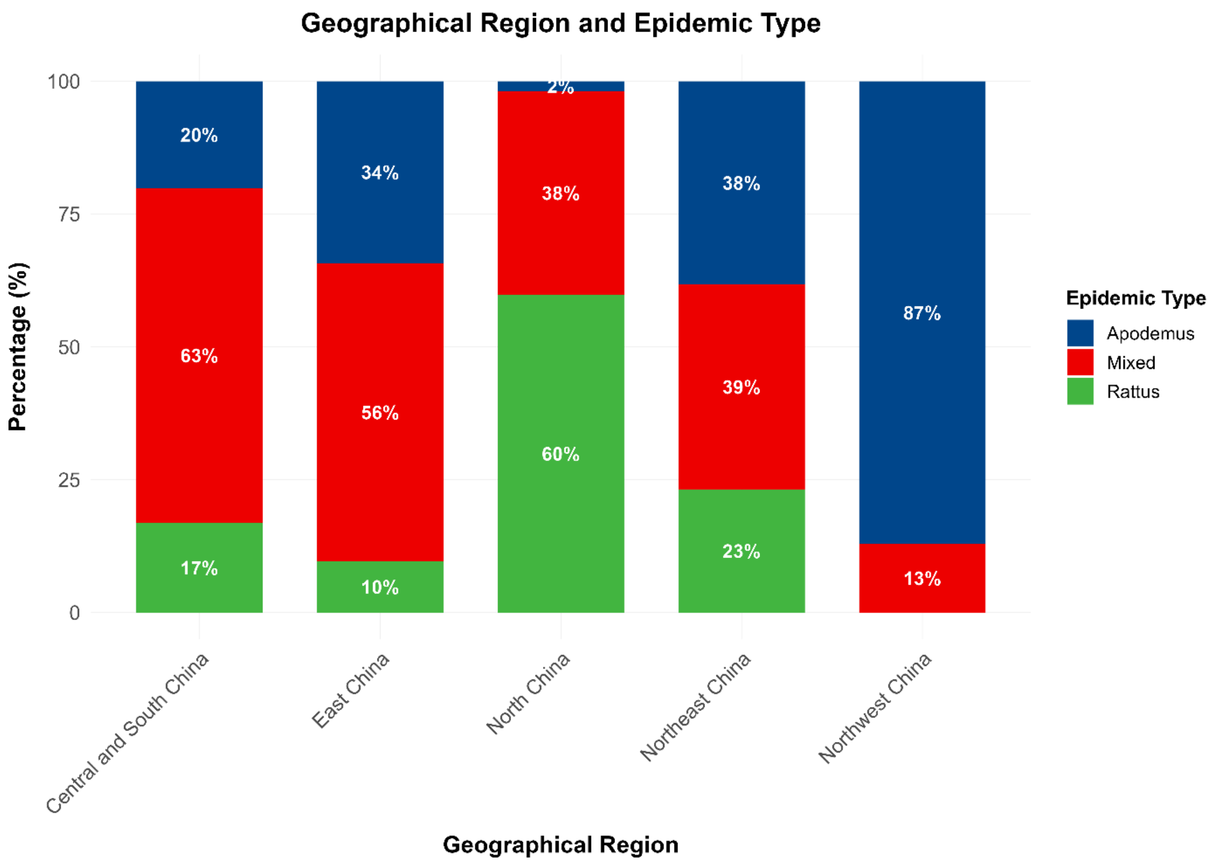

Figure S6. Composition of epidemic types in HFRS in different geographical regions.

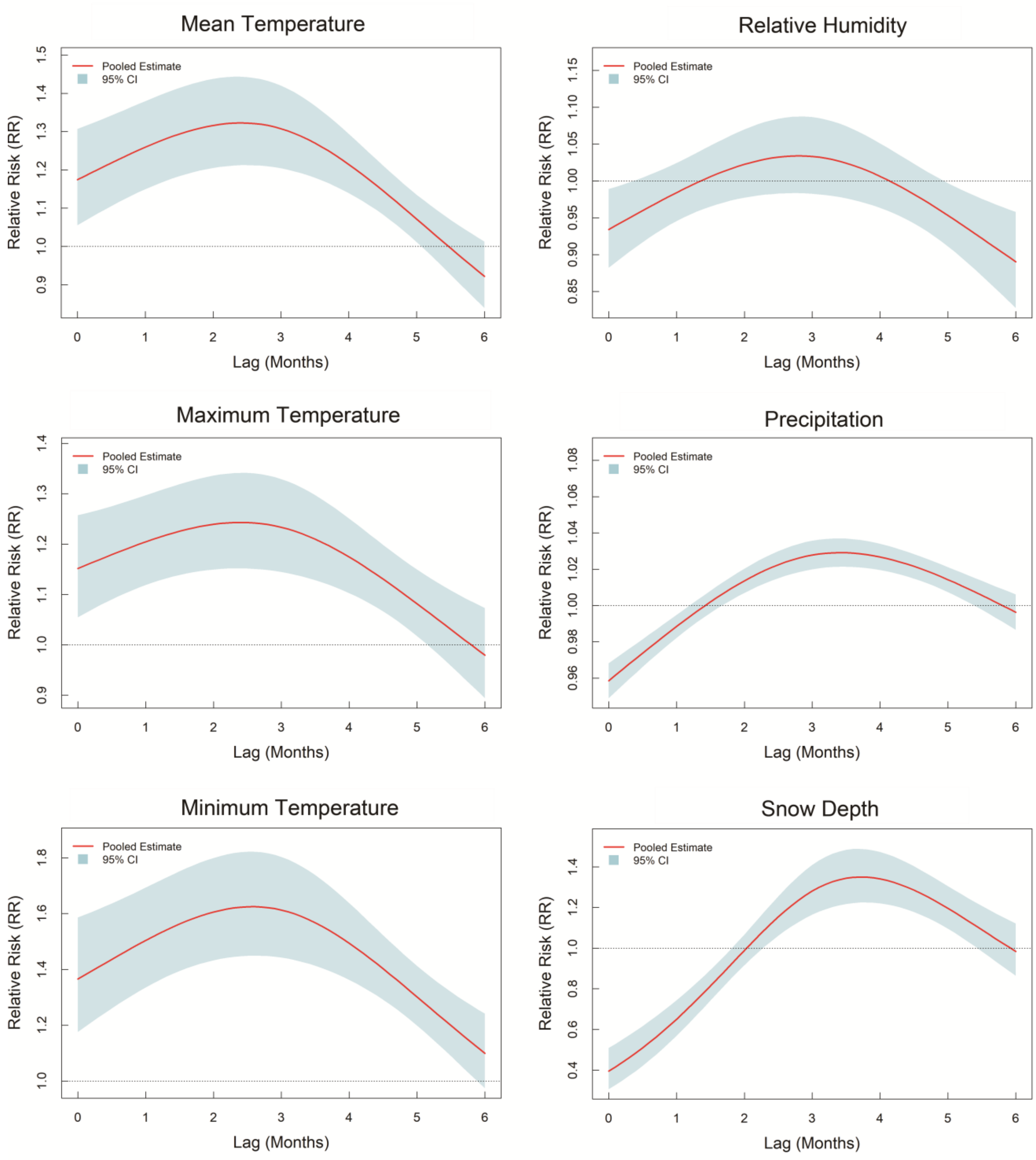

Figure S7. Lag effects of meteorological factors on the incidence of HFRS.

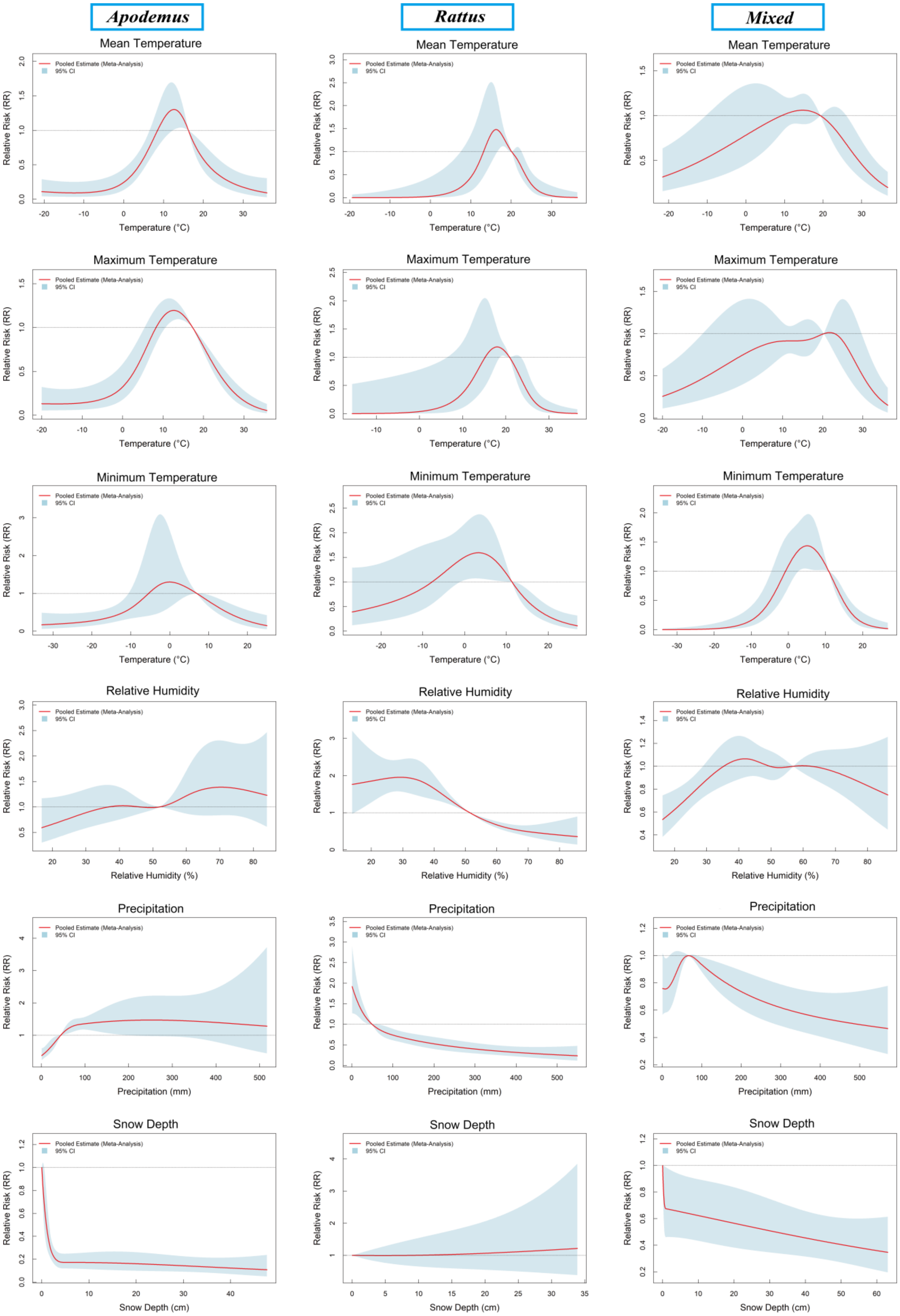

Figure S8. Nonlinear effects of meteorological factors in regions with different epidemic types.

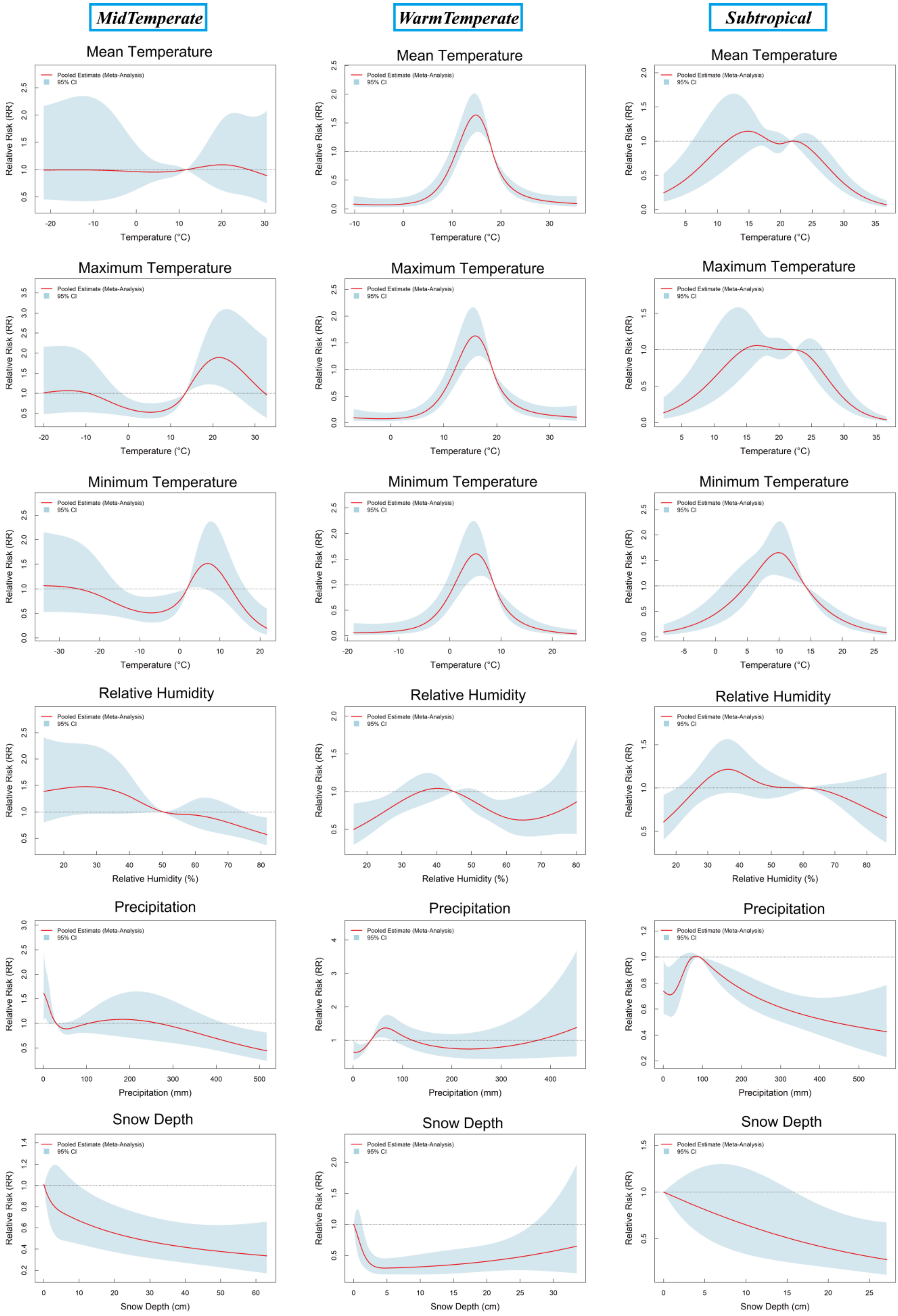

Figure S9. Nonlinear effects of meteorological factors in different climate zones.

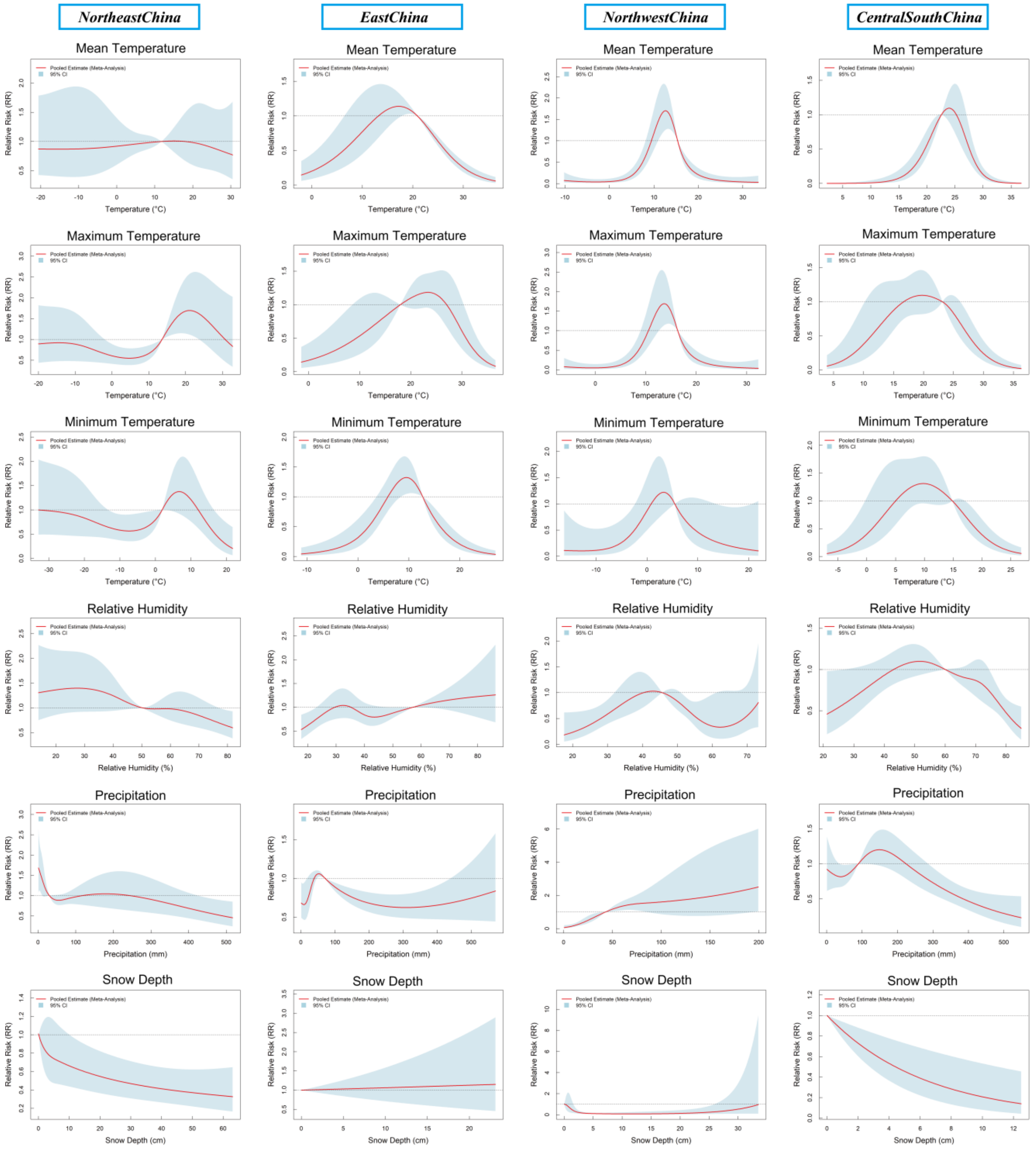

Figure S10. Nonlinear effects of meteorological factors in different geographical regions.

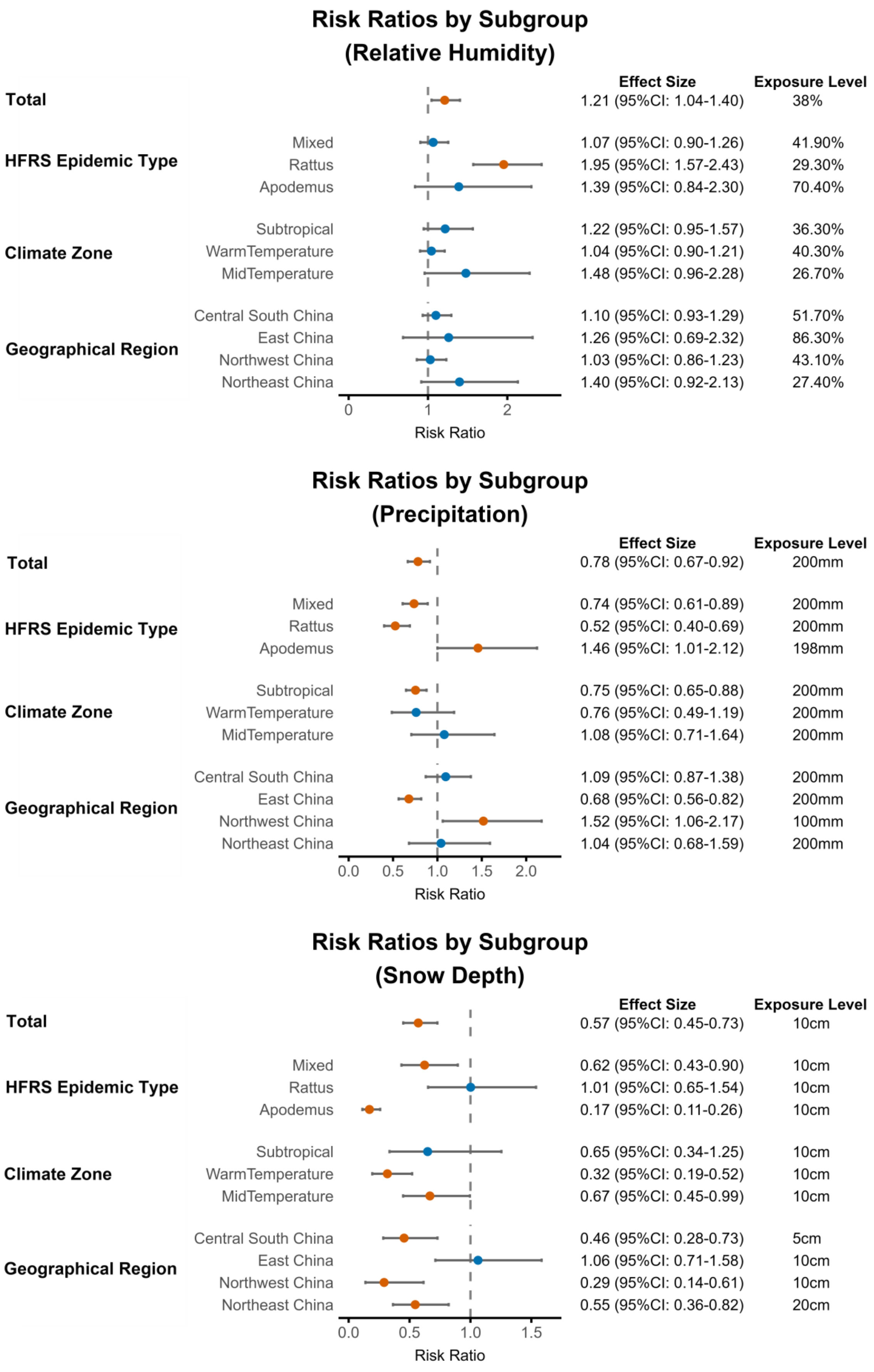

Figure S11. Differences in effects of relative humidity, precipitation and snow depth in different regions.
（Note: Red dots represent statistically significant results, and blue dots represent results that are not statistically significant.）

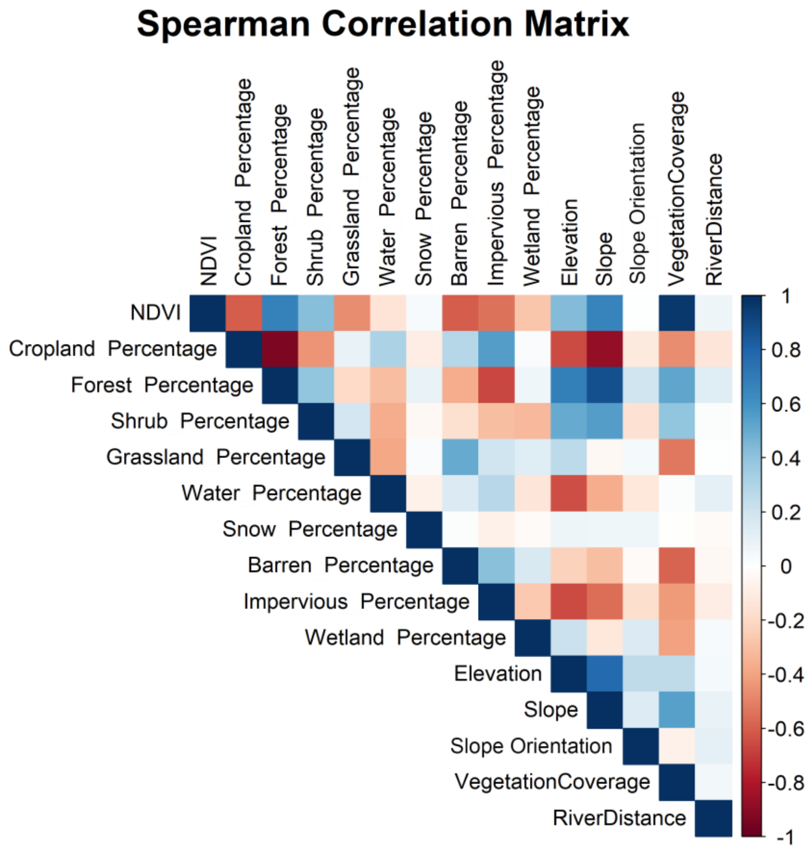

Figure S12. Correlations of environmental factors.

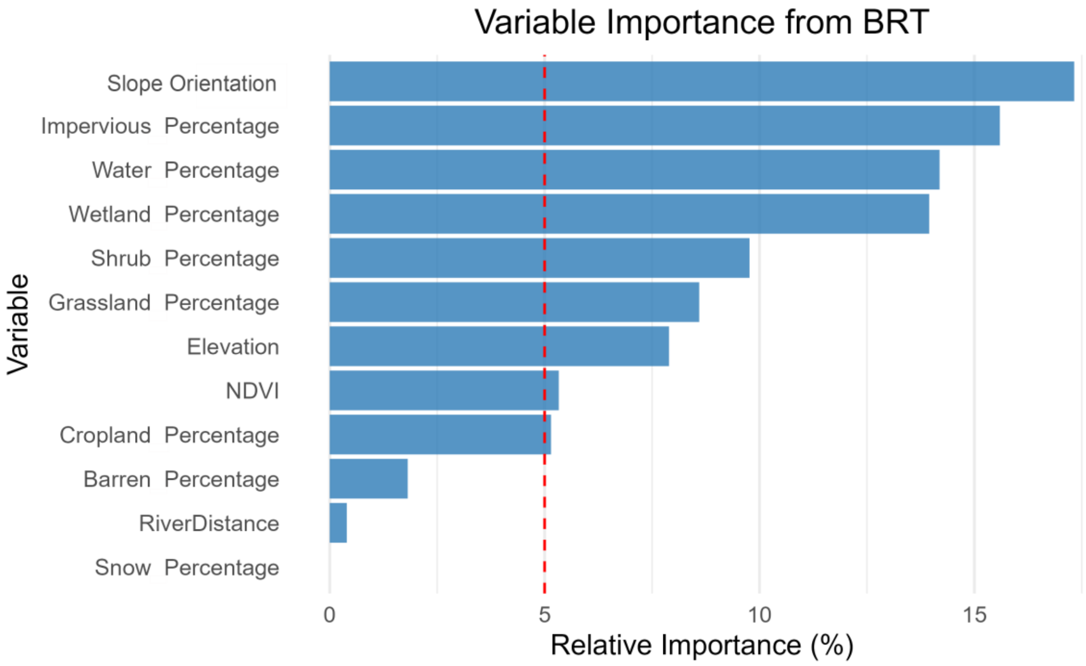

Figure S13. Relative importance of environmental factors.

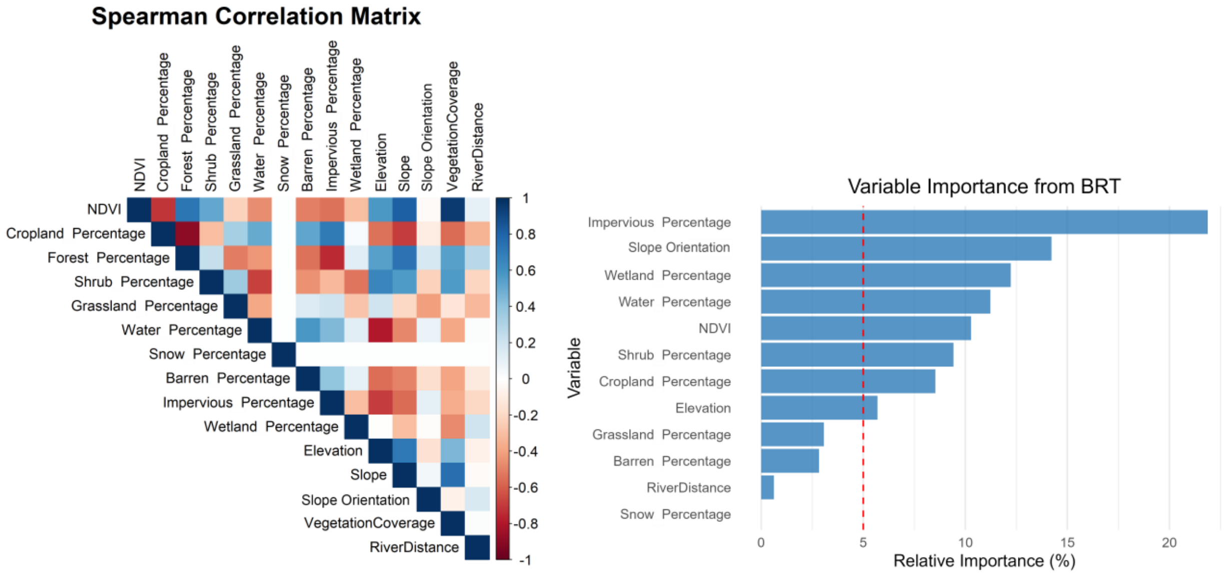

Figure S14. Correlation and importance of environmental factors in *Apodemus*-Type regions.

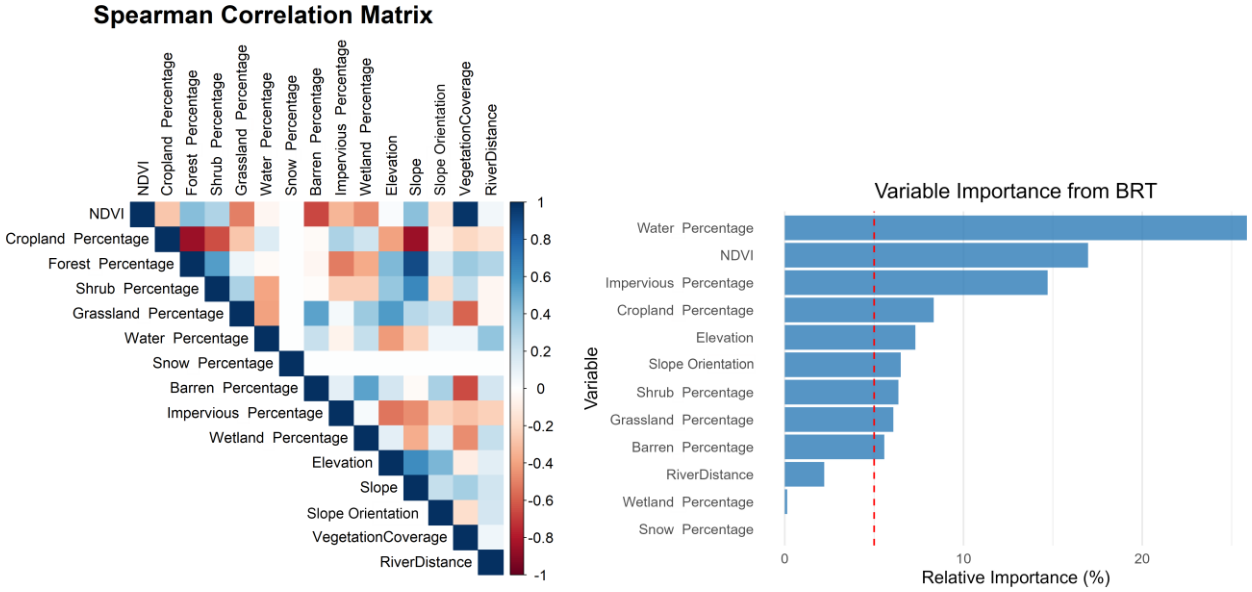

Figure S15. Correlation and importance of environmental factors in *Rattus*-Type regions.

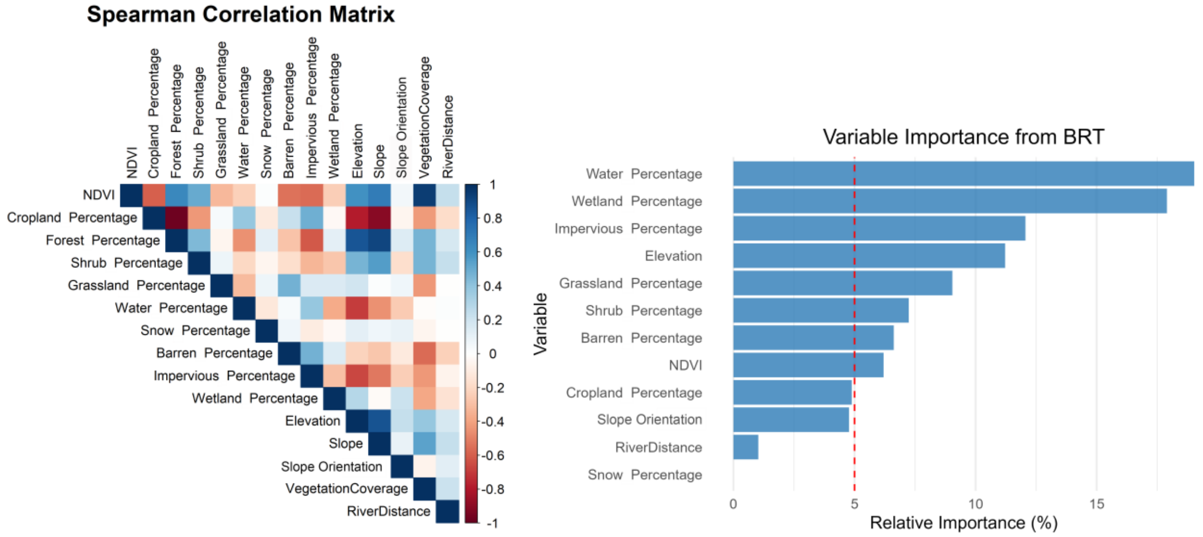

Figure S16. Correlation and importance of environmental factors in Mixed-Type regions.

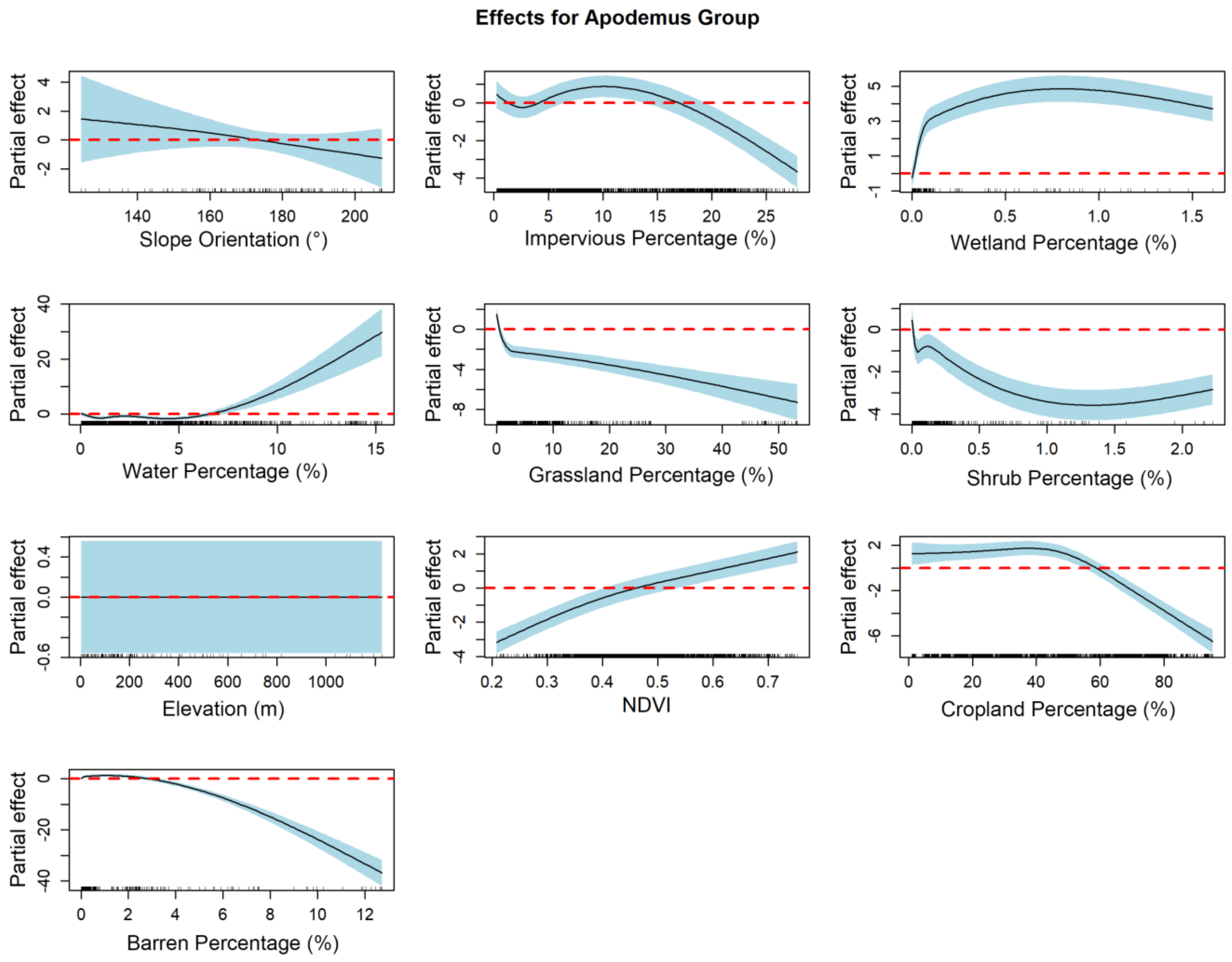

Figure S17. Effects of environmental factors in *Apodemus*-Type regions.

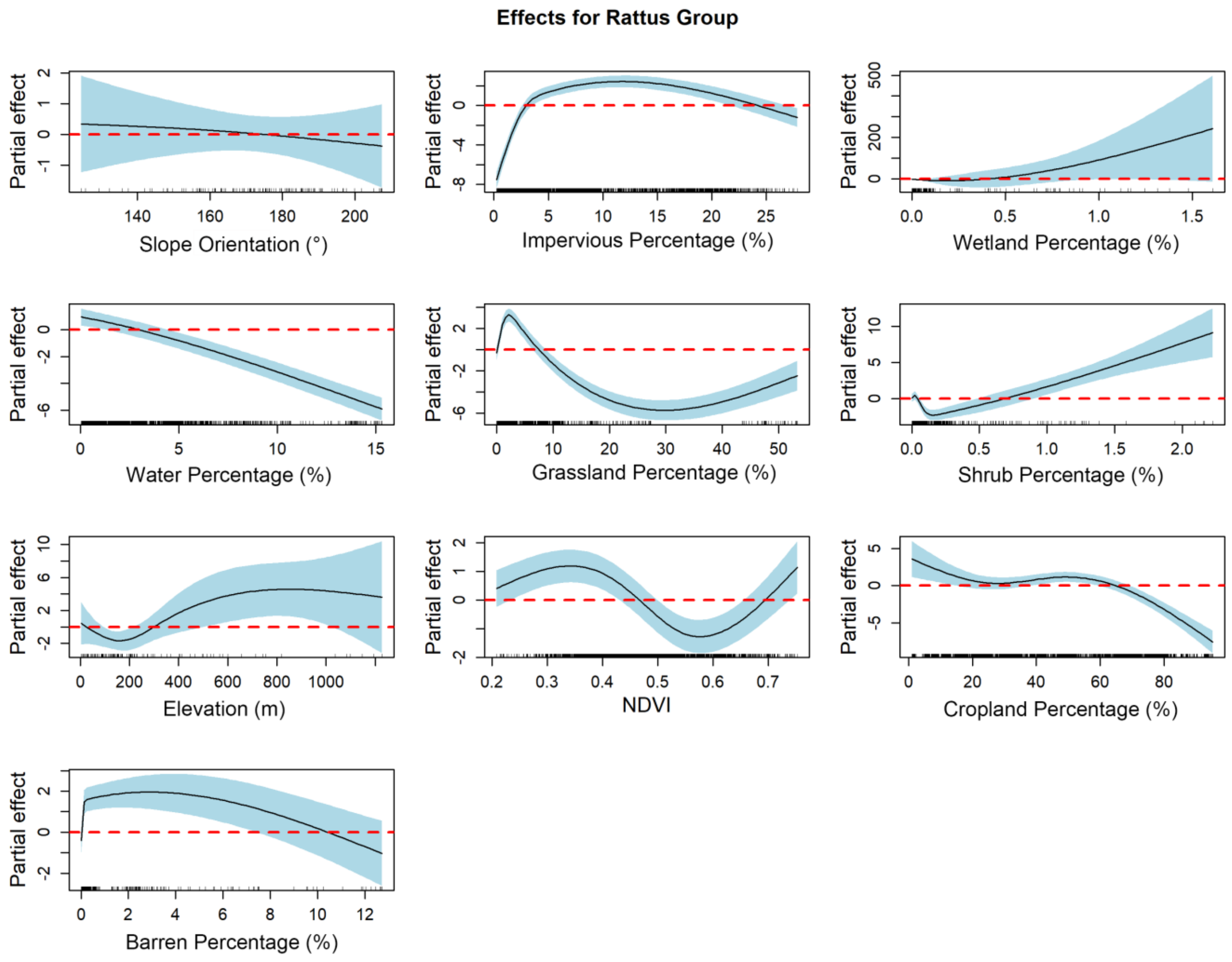

Figure S18. Effects of environmental factors in *Rattus*-Type regions.

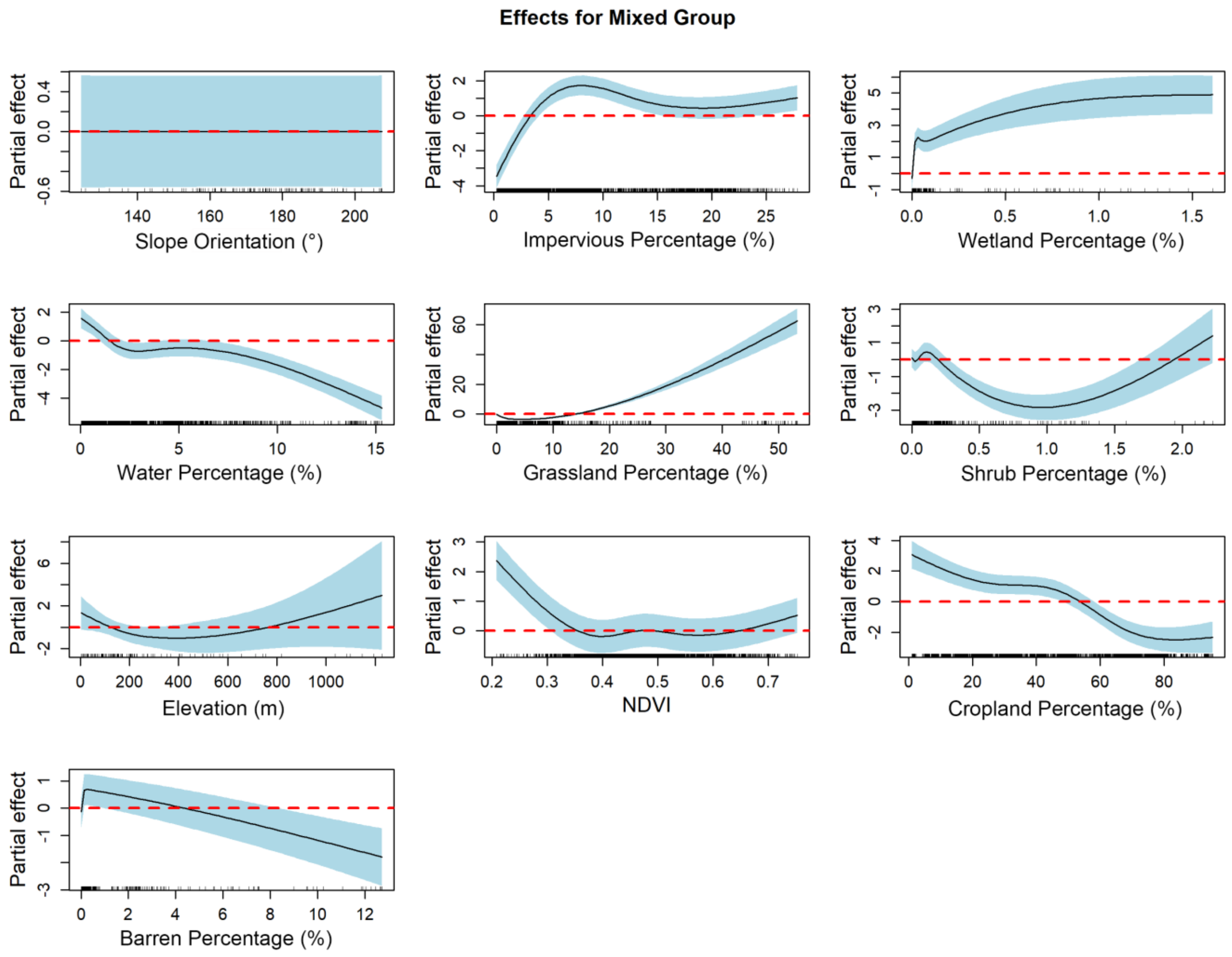

Figure S19. Effects of environmental factors in Mixed-Type regions.

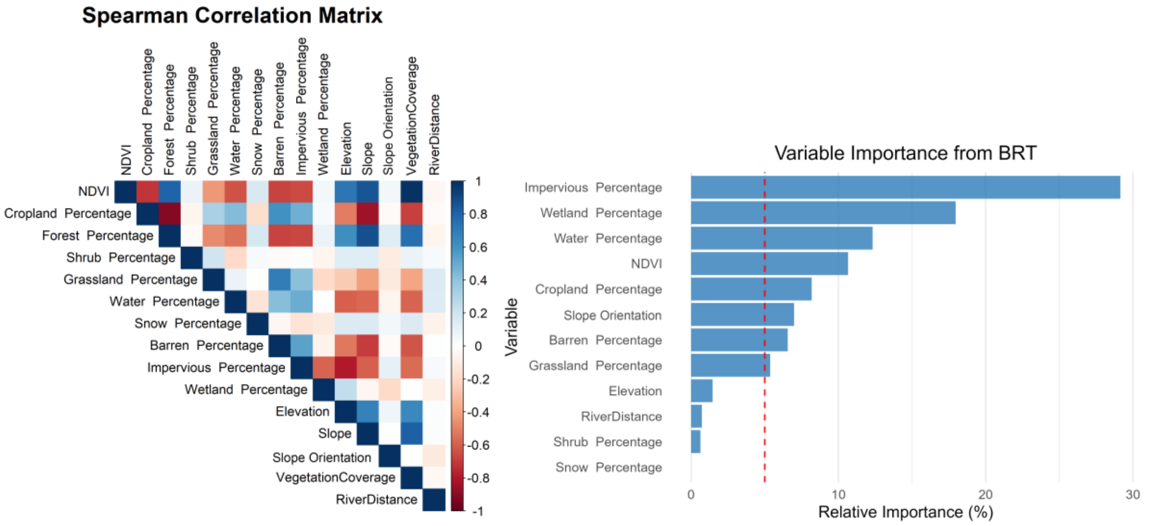

Figure S20. Correlation and importance of environmental factors in mid-temperate zone.

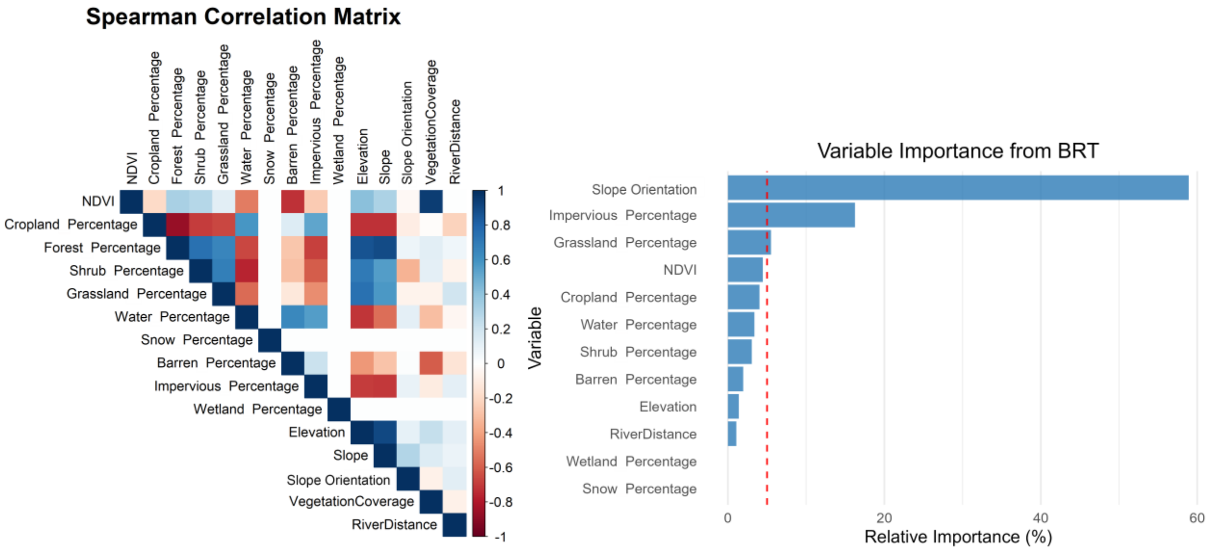

Figure S21. Correlation and importance of environmental factors in warm temperate zone.

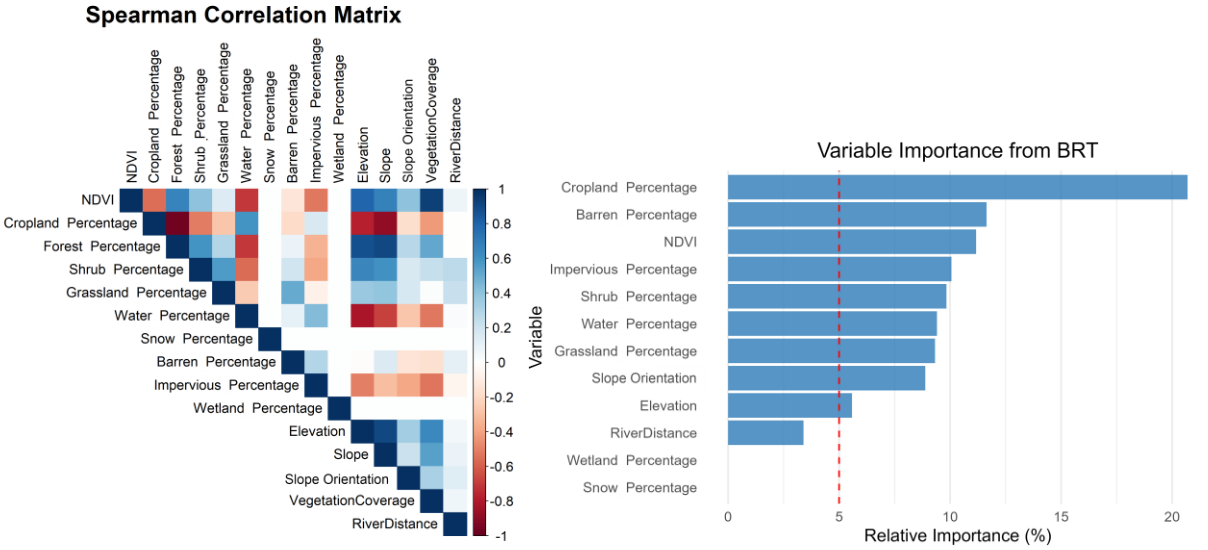

Figure S22. Correlation and importance of environmental factors in subtropical zone.

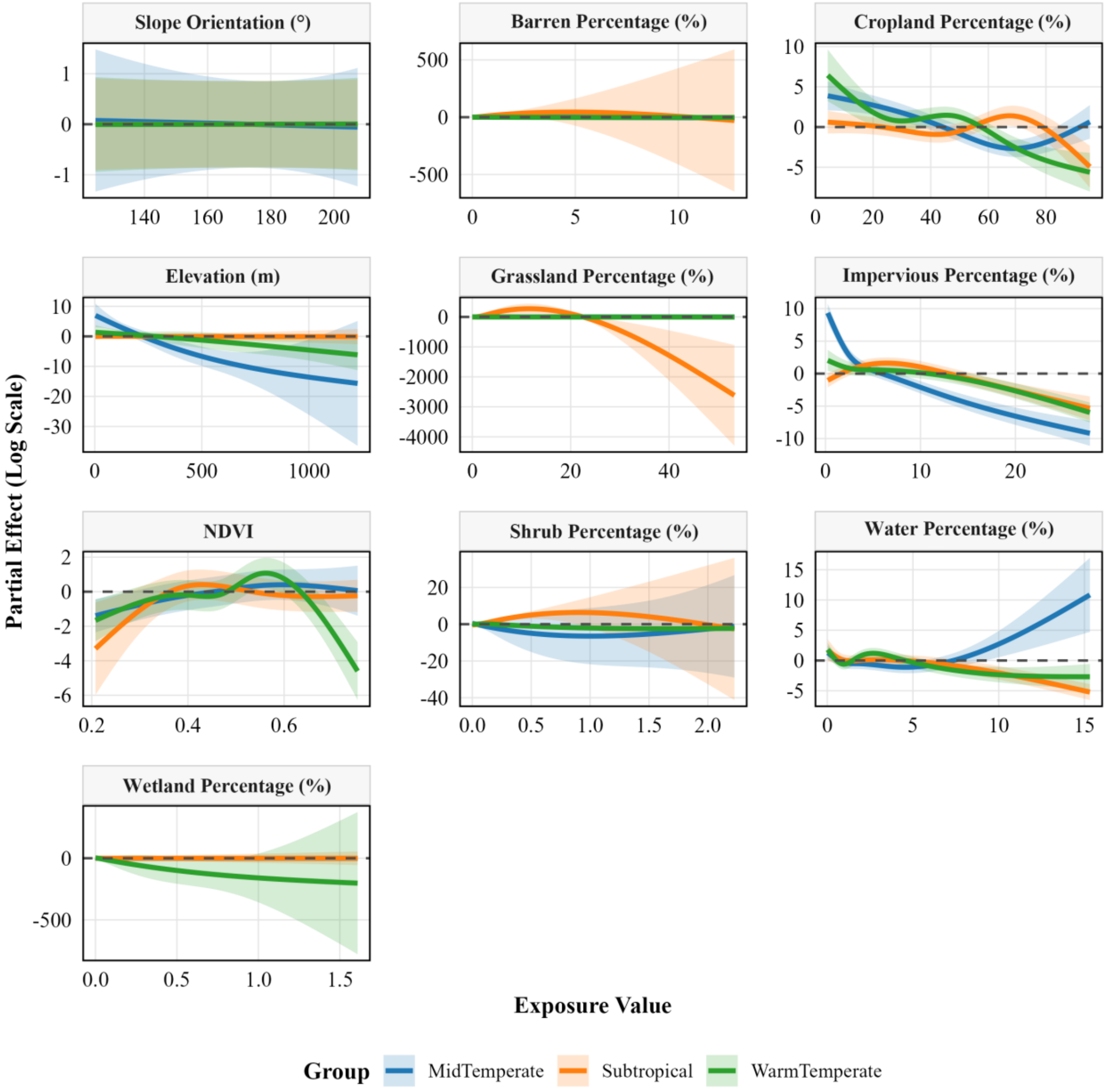

Figure S23. Differences in the effects of environmental factors in different climate zones.

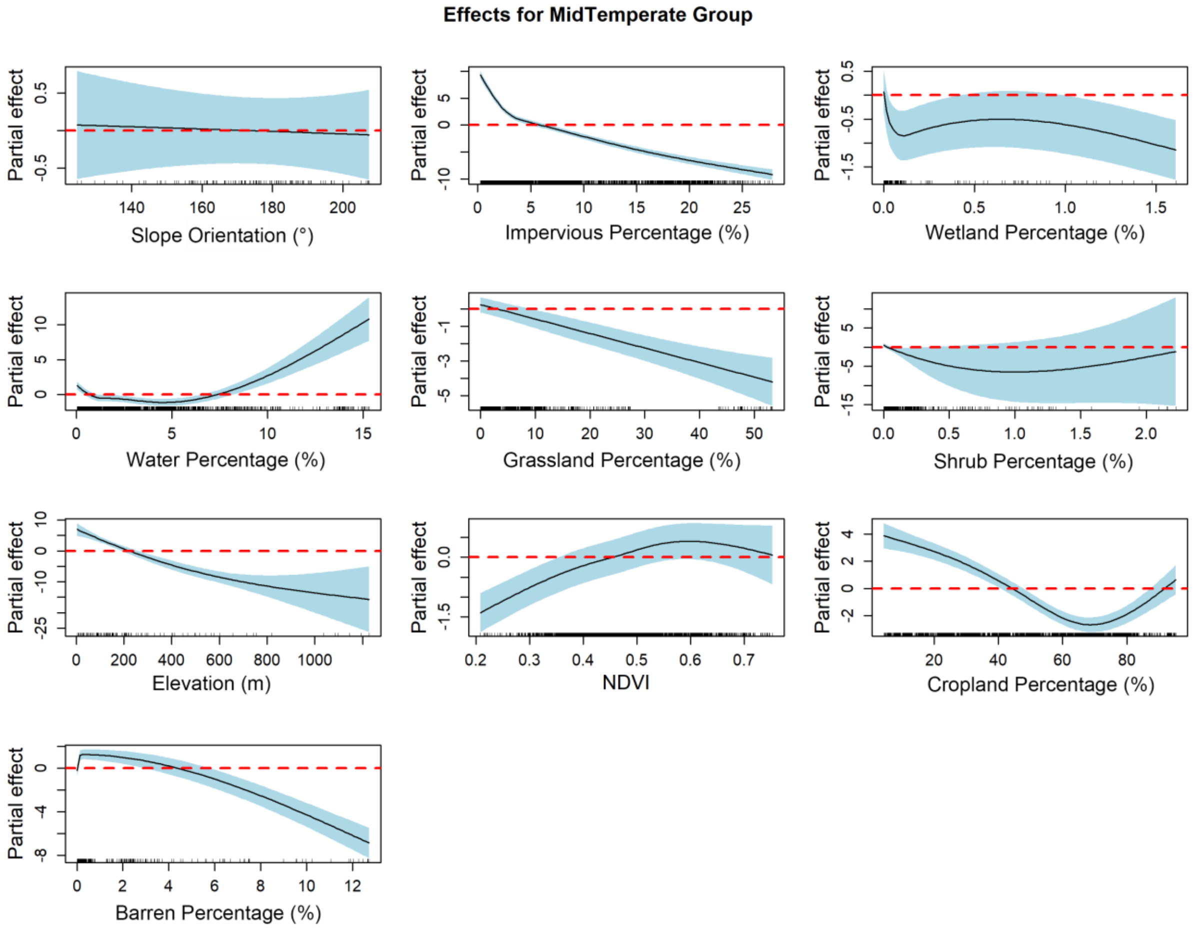

Figure S24. Effects of environmental factors in mid-temperate zone.

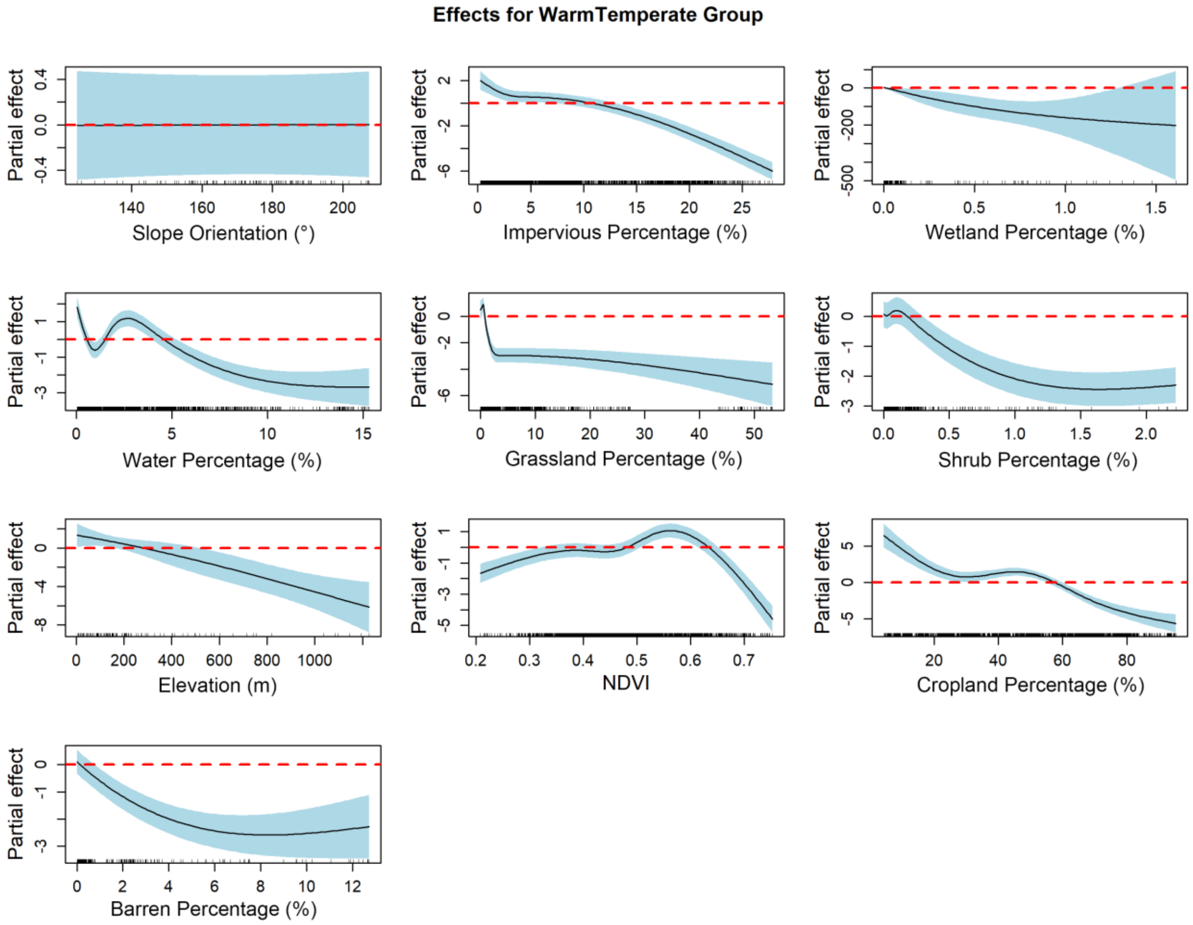

Figure S25. Effects of environmental factors in warm temperate zone.

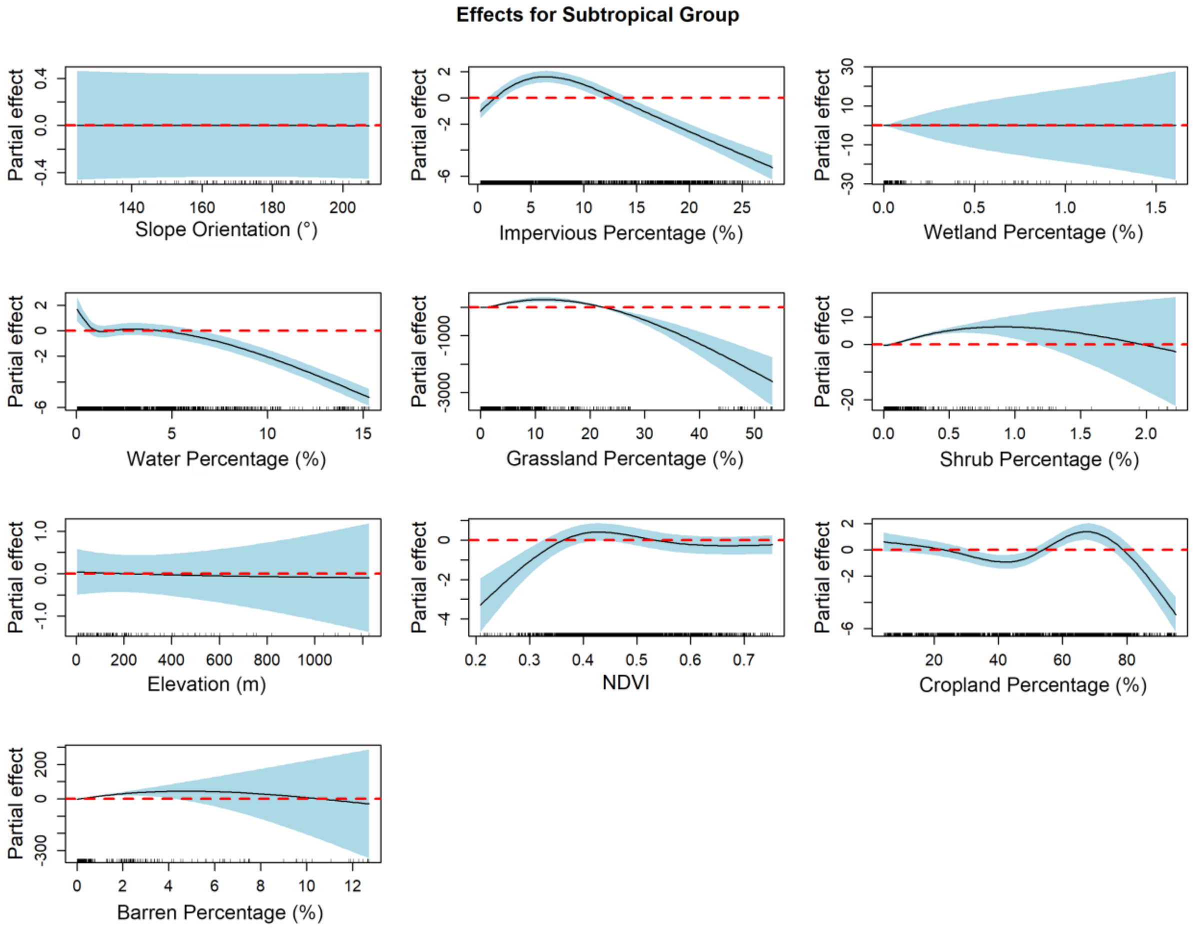

Figure S26. Effects of environmental factors in subtropical zone.

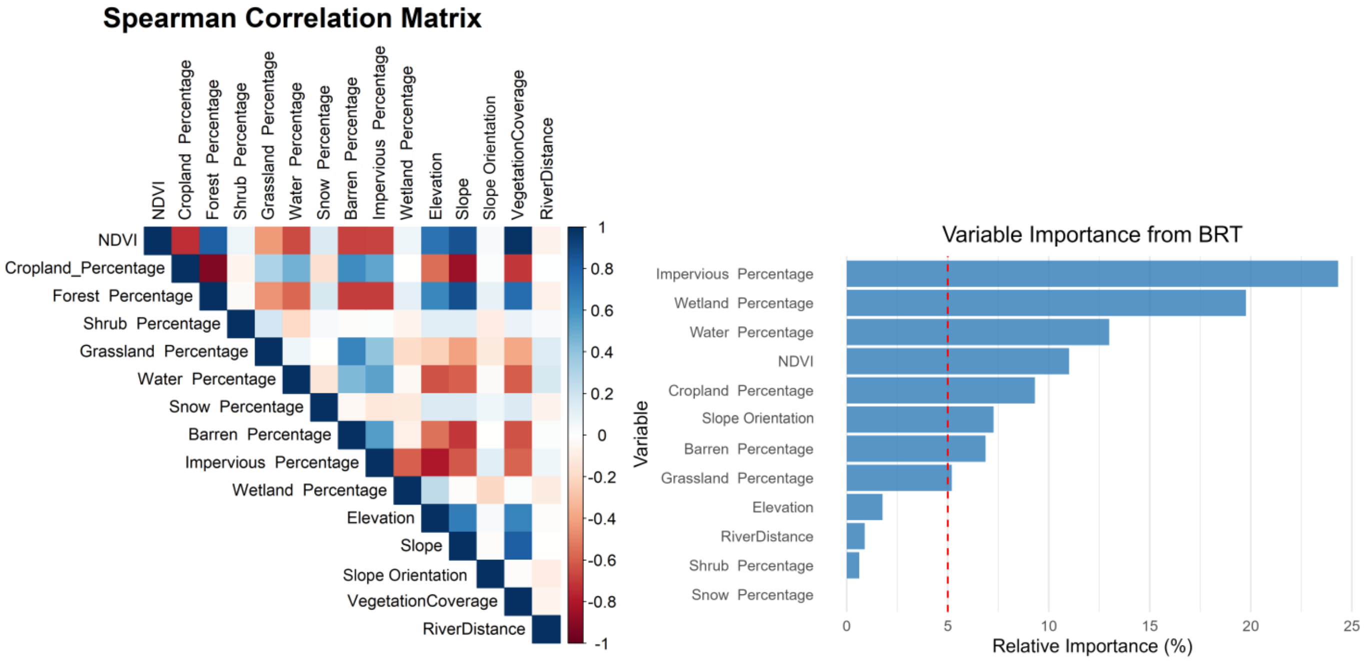

Figure S27. Correlation and importance of environmental factors in Northeast China.

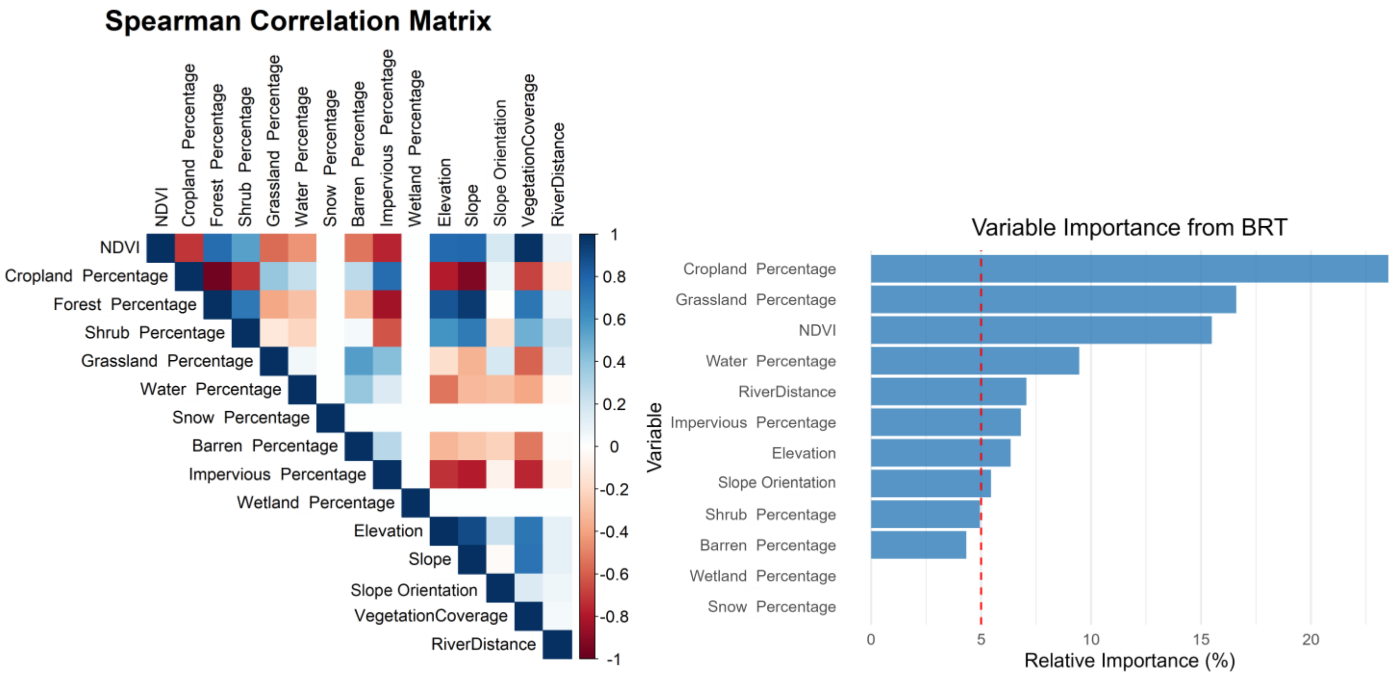

Figure S28. Correlation and importance of environmental factors in East China.

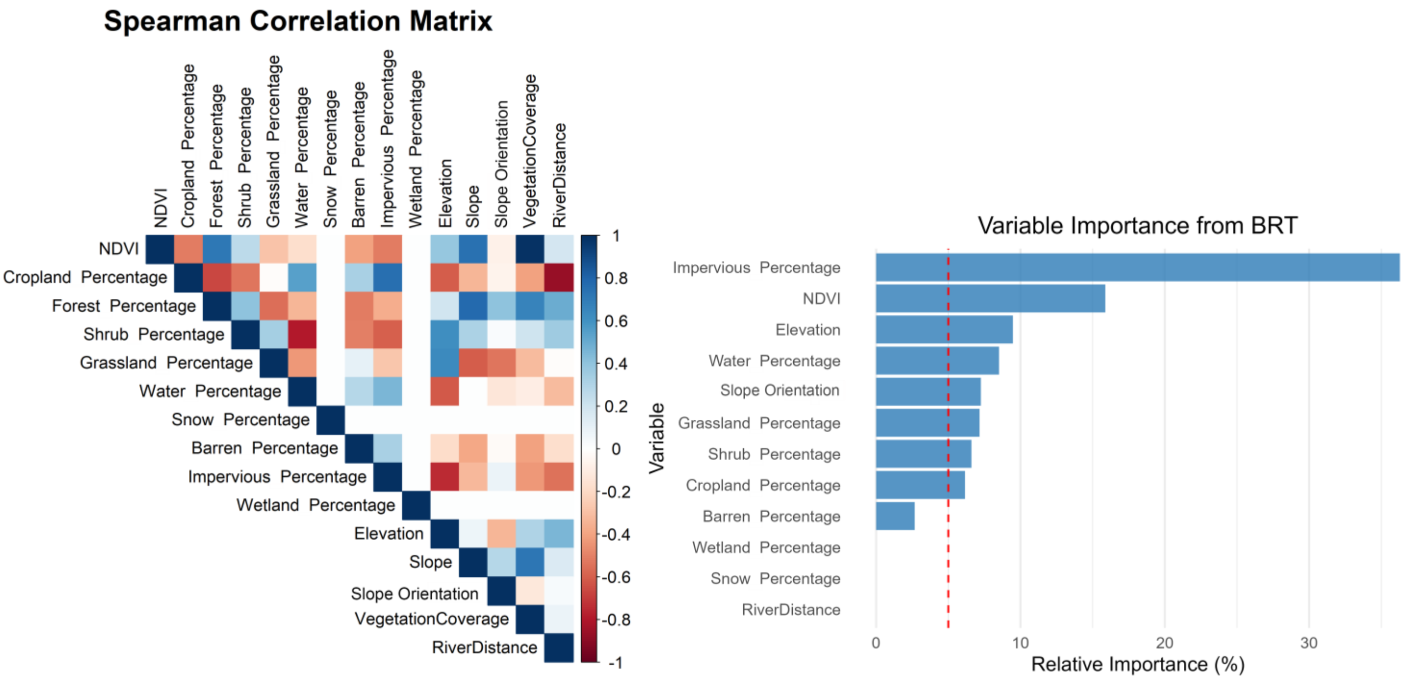

Figure S29. Correlation and importance of environmental factors in Northwest China.

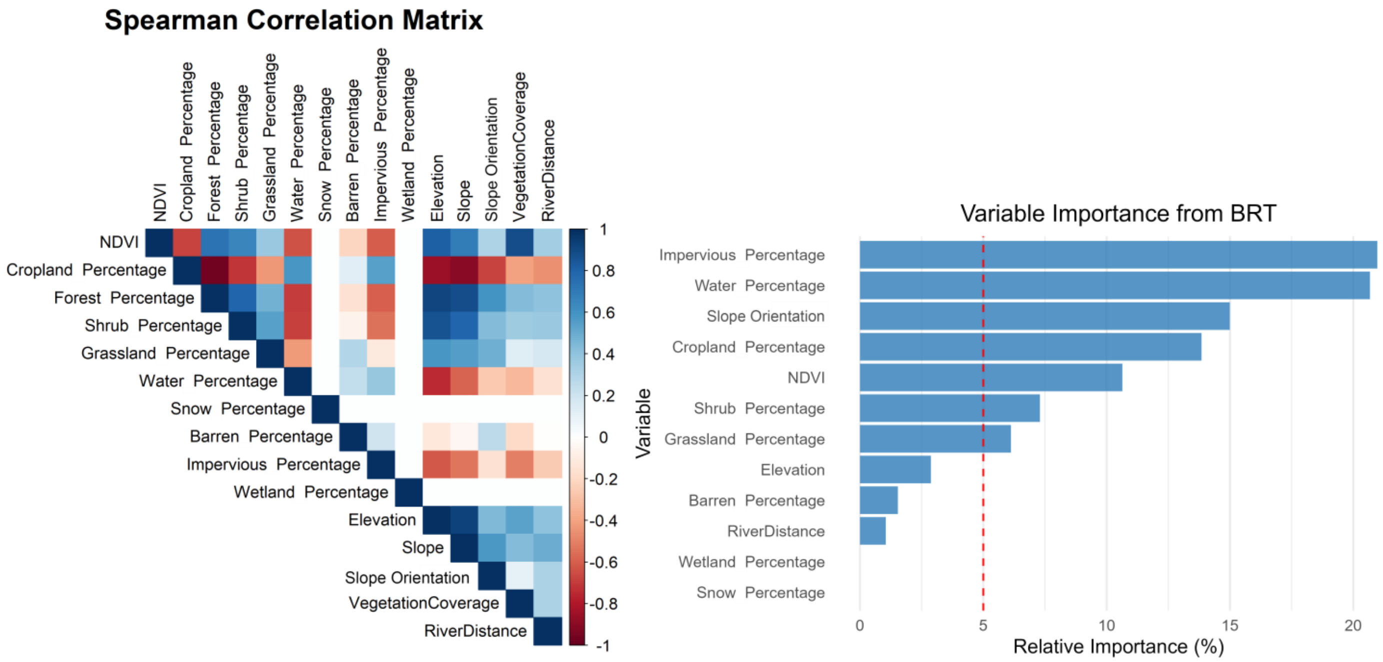

Figure S30. Correlation and importance of environmental factors in Central South China.

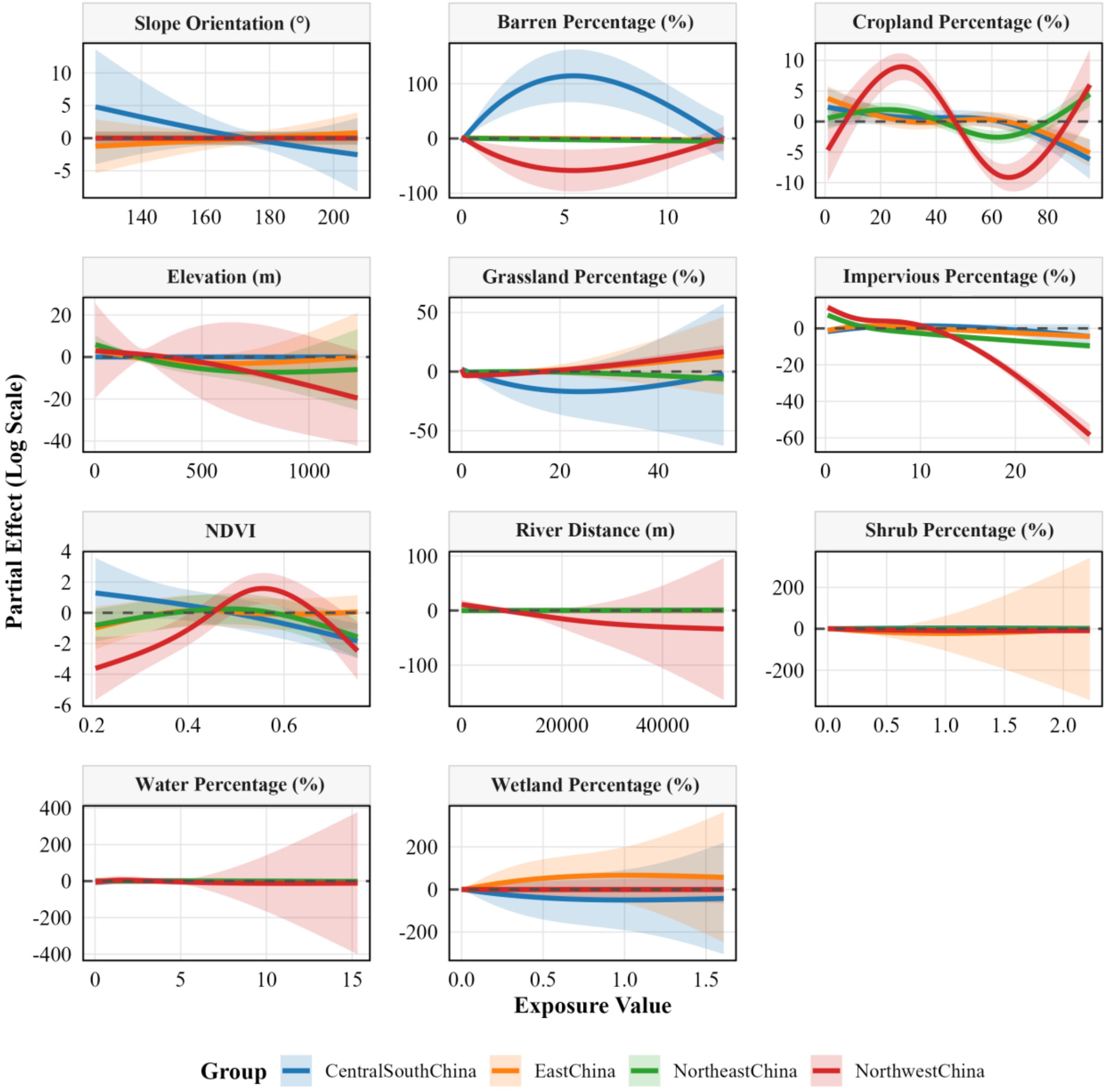

Figure S31. Differences in the effects of environmental factors in different geographical regions.

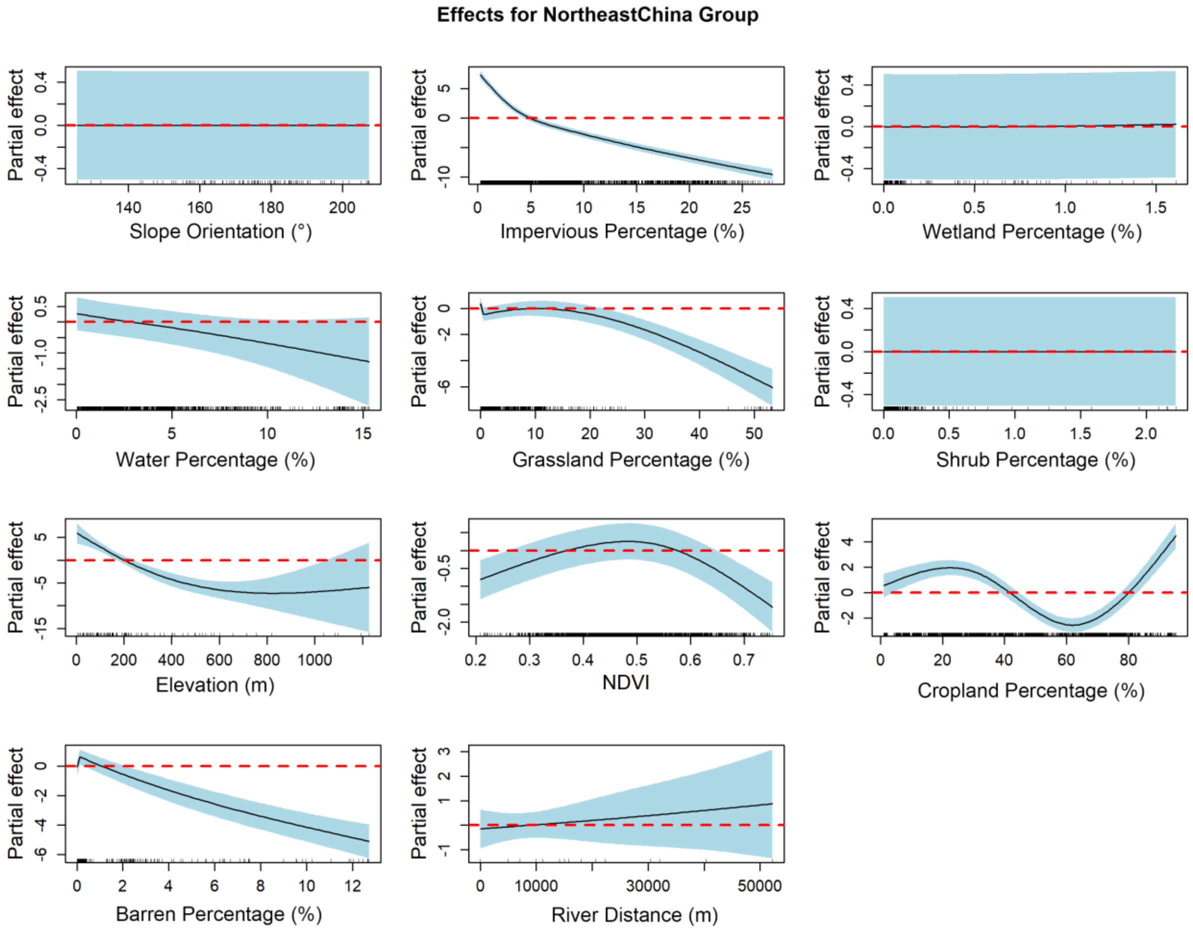

Figure S32. Effect of environmental factors in Northeast China.

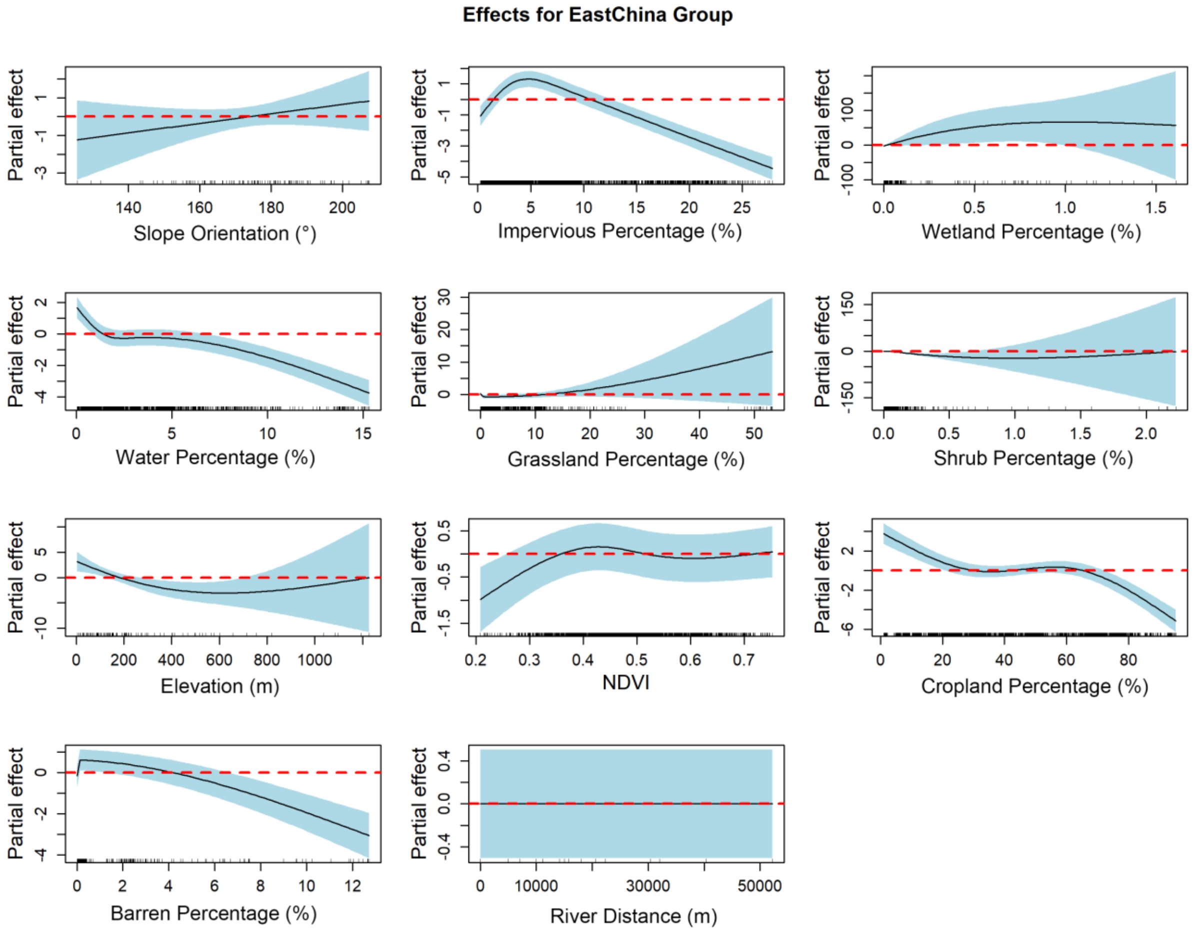

Figure S33. Effects of environmental factors in East China.

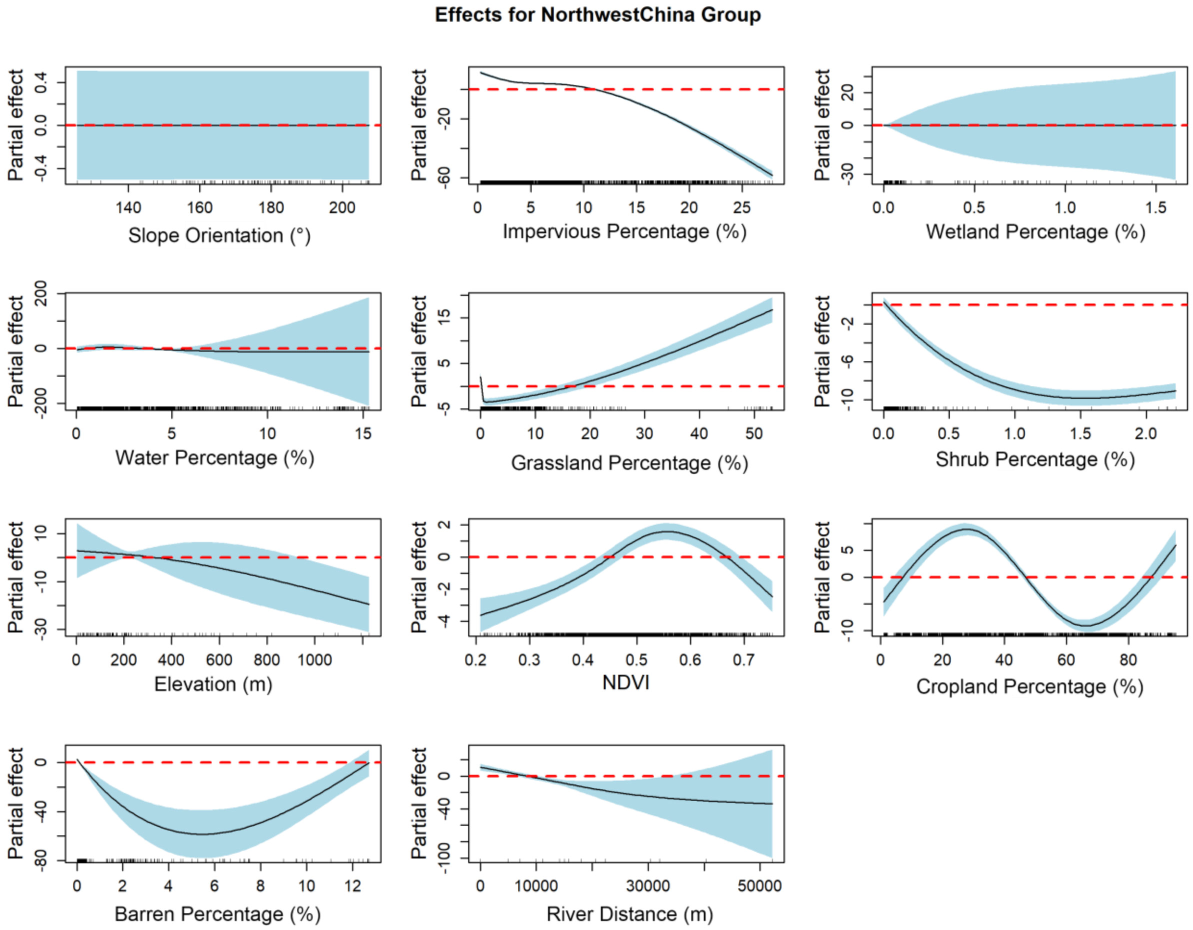

Figure S34. Effects of environmental factors in Northwest China.

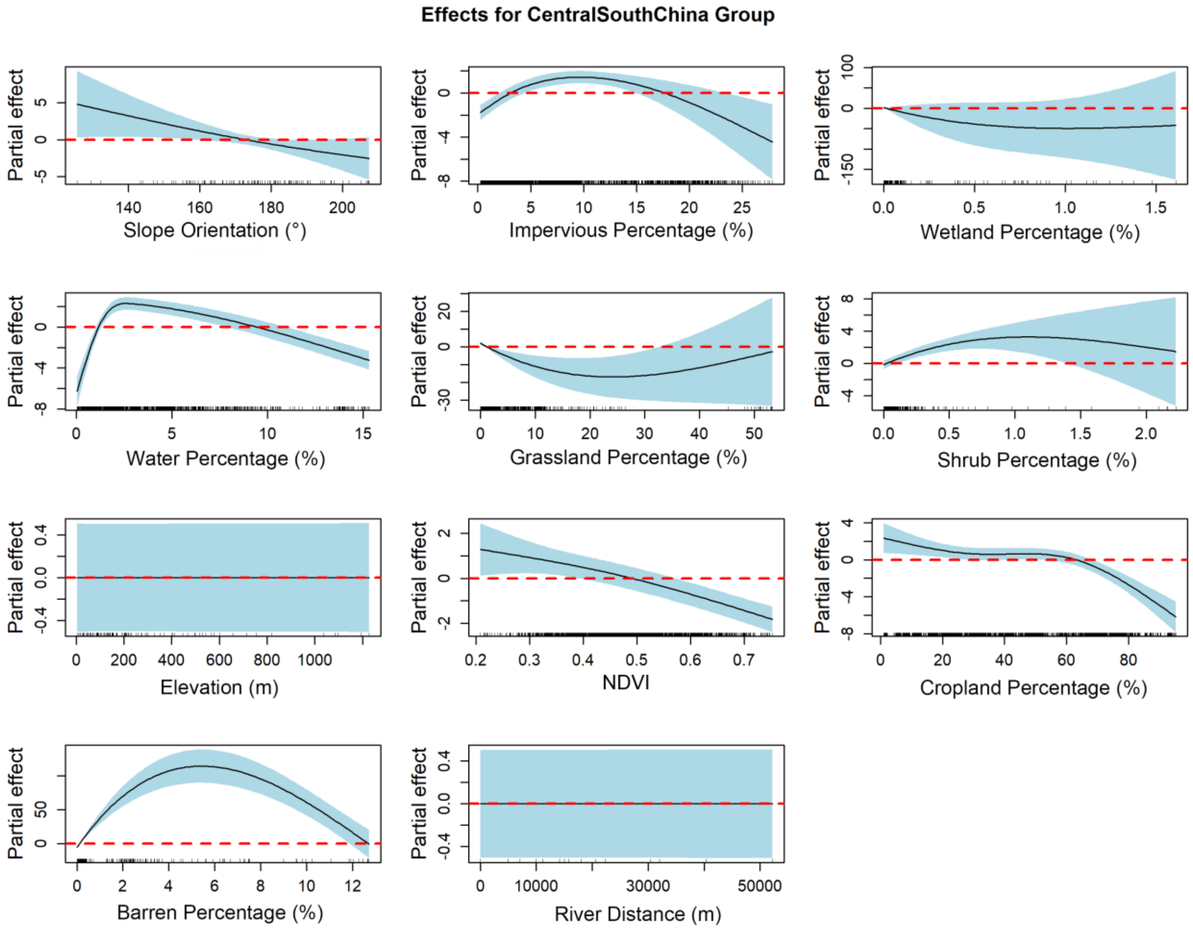

Figure S35. Effects of environmental factors in Central South China.

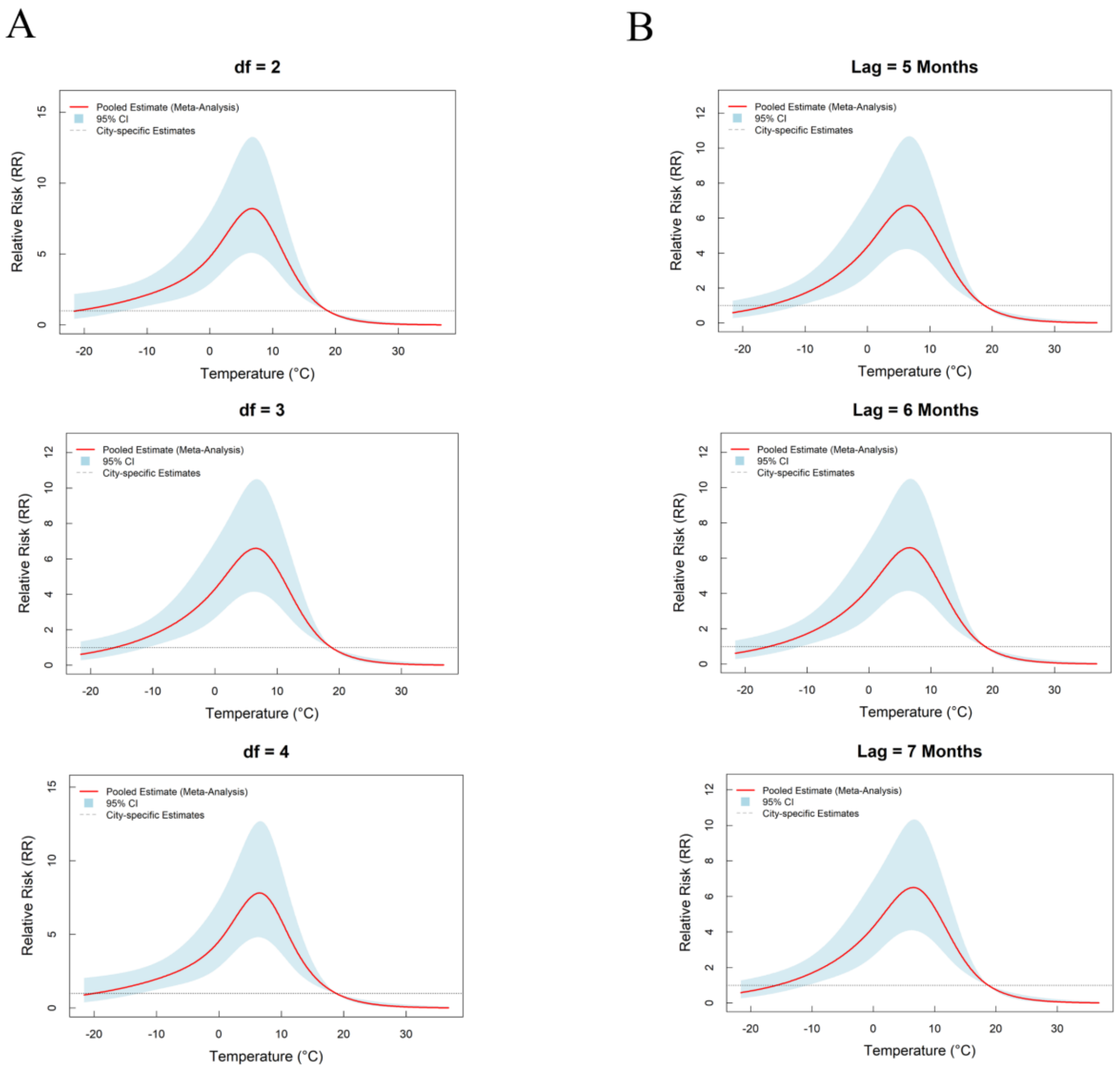

Figure S36. Sensitivity analysis of DLNM.

Note: A: changing the lag periods (lag = 6, 5, 7); B: changing the degrees of freedom (df = 2, 3, 4).

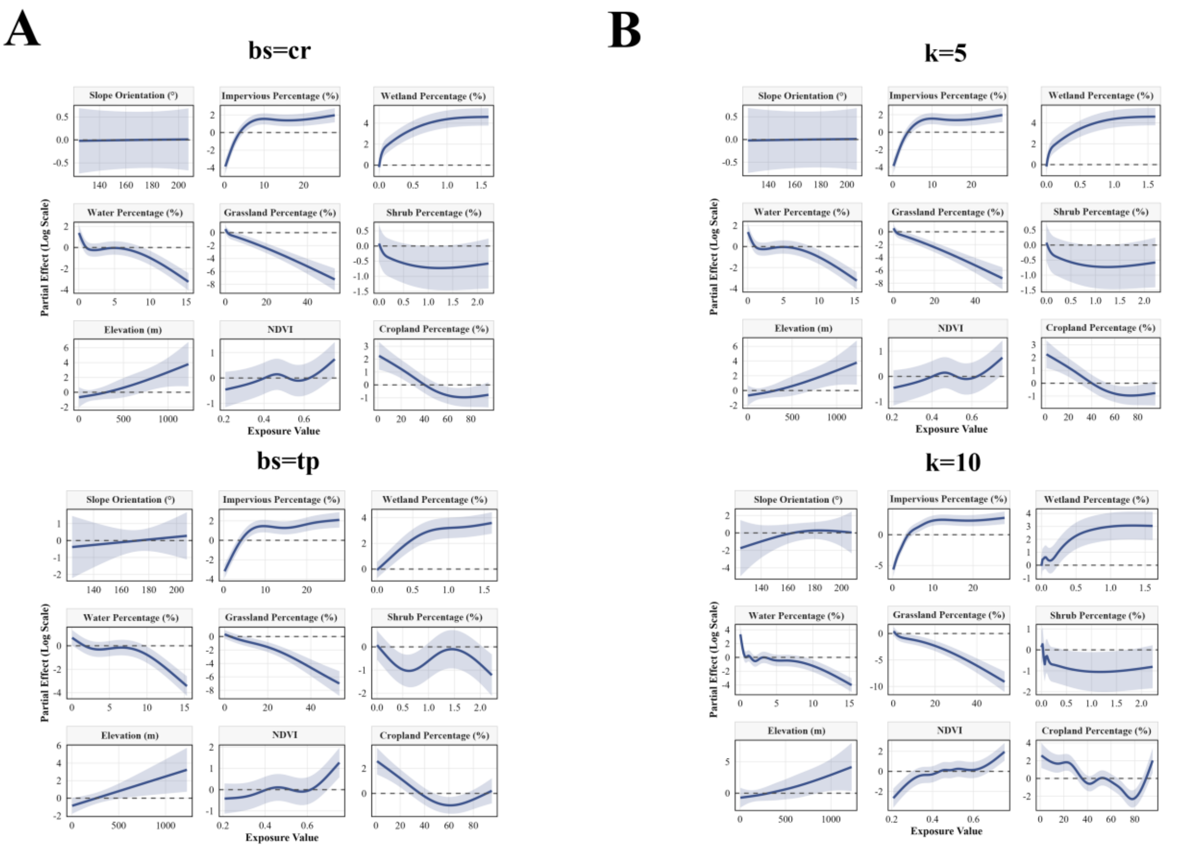

Figure S37. Sensitivity analysis of GAMM.

Note: A: changing the spline function (cubic spline and thin plate spline); B: changing the smoothing parameter (k = 5, 10).

References

1. Lv CL, Tian Y, Qiu Y, et al. Dual seasonal pattern for hemorrhagic fever with renal syndrome and its potential determinants in China. *Sci Total Environ.* 2023;859(Pt 2):160339. doi:10.1016/j.scitotenv.2022.160339.
2. Wang Y, Zhang C, Gao J, et al. Spatiotemporal trends of hemorrhagic fever with renal syndrome (HFRS) in China under climate variation. *PNAS.* 2024;121(4):e2312556121. doi:10.1073/pnas.2312556121.
3. Jixi G, Yuanli S, Hongwei Z, et al. China regional 250m normalized difference vegetation index data set (2000-2023). Accessed April 15,2025. https://dx.doi.org/10.11888/Terre.tpdc.300328.
4. Gao Jixi, Yuanli S, Hongwei Z, et al. China regional 250m fractional vegetation cover data set (2000-2023). Accessed March 4,2025. https://dx.doi.org/10.11888/Terre.tpdc.300330.
5. Yang J, Huang X. The 30 m annual land cover datasets and its dynamics in China from 1985 to 2023. Accessed March 4,2025. https://dx.doi.org/10.5281/zenodo.12779975.
6. Guoan T. Digital elevation model of China (1KM). Accessed February 1,2025. https://data.tpdc.ac.cn/zh-hans/data/12e91073-0181-44bf-8308-c50e5bd9a734.
7. XinLiang X. Dataset of watershed and river network in China based on DEM. Accessed April 15,2025. https://dx.doi.org/10.12078/2018060101.
